# Combining Experimental Sorption Parameters with QSAR to Predict Neonicotinoid and Transformation Product Sorption to Carbon Nanotubes and Granular Activated Carbon

**DOI:** 10.1021/acsestwater.1c00492

**Published:** 2022-01-05

**Authors:** Danielle T. Webb, Matthew R. Nagorzanski, David M. Cwiertny, Gregory H. LeFevre

**Affiliations:** † Department of Civil & Environmental Engineering, University of Iowa, 4105 Seamans Center, Iowa City, Iowa 52242, United States; ‡ IIHR—Hydroscience & Engineering, 100 C. Maxwell Stanley Hydraulics Laboratory, Iowa City, Iowa 52242, United States; § Center for Health Effects of Environmental Contamination, University of Iowa, 455 Van Allen Hall, Iowa City, Iowa 52242, United States; ∥ Public Policy Center, University of Iowa, 310 South Grand Avenue, 209 South Quadrangle, Iowa City, Iowa 52242, United States

**Keywords:** neonicotinoids, sorption, carbon nanotubes, granular activated carbon, QSAR, multiple linear regression, prediction

## Abstract

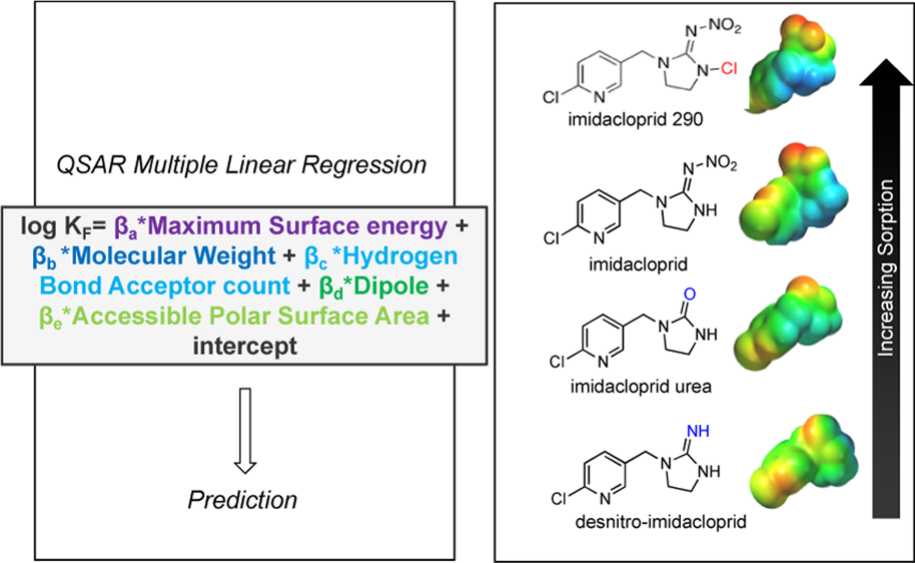

We recently discovered that transformation of the neonicotinoid insecticidal pharmacophore alters sorption propensity to activated carbon, with products adsorbing less than parent compounds. To assess the environmental fate of novel transformation products that lack commercially available standards, researchers must rely on predictive approaches. In this study, we combined computationally derived quantitative structure–activity relationship (QSAR) parameters for neonicotinoids and neonicotinoid transformation products with experimentally determined Freundlich partition constants (log *K*_F_ for sorption to carbon nanotubes [CNTs] and granular activated carbon [GAC]) to model neonicotinoid and transformation product sorption. QSAR models based on neonicotinoid sorption to functionalized/nonfunctionalized CNTs (used to generalize/simplify neonicotinoid-GAC interactions) were iteratively generated to obtain a multiple linear regression that could accurately predict neonicotinoid sorption to CNTs using internal and external validation (within 0.5 log units of the experimentally determined value). The log *K*_F,CNT_ values were subsequently related to log *K*_F,GAC_ where neonicotinoid sorption to GAC was predicted within 0.3 log-units of experimentally determined values. We applied our neonicotinoid-specific model to predict log *K*_F,GAC_ for a suite of novel neonicotinoid transformation products (i.e., formed via hydrolysis, biotransformation, and chlorination) that do not have commercially available standards. We present this modeling approach as an innovative yet relatively simple technique to predict fate of highly specialized/unique polar emerging contaminants and/or transformation products that cannot be accurately predicted via traditional methods (e.g., pp-LFER), and highlights molecular properties that drive interactions of emerging contaminants.

## Abstract 2

A neonicotinoid-tailored QSAR model with internal/external validation successfully predicted sorption to activated carbon and outperformed traditional pp-LFER models, while enabling predictive power for novel transformation products.

## Introduction

Neonicotinoids are among the most widely used insecticides in the world with applications in agriculture, forestry, home pest control, and pet flea and tick preventatives.^1−5^ Due to their widespread use and hydrophilic nature (log *K*_ow_ −0.64 to 1.26),^6^ neonicotinoids have become ubiquitous in natural (ground and surface waters) and engineered systems (drinking water and wastewater treatment plants) throughout the U.S. with detections between <1 and ∼100 μg/L.^1,3,4,7−19^ Although their widespread occurrence is now recognized, there are a limited number of studies that have reported the presence and/or fate of neonicotinoid transformation products (formed via microbial degradation,^20−24^ chlorination,^16^ hydrolysis,^15,16,25,26^ and photolysis^25^) in these systems. Because some neonicotinoid transformation products can have altered toxicities (e.g., desnitro-imidacloprid is >300× more toxic to mammals than parent imidacloprid),^27−31^ a deeper understanding of the fate of novel transformation products in natural and engineered systems is critical.

Partitioning coefficients are a common metric for assessing contaminant fate in the environment (e.g., octanol–water, air–water, solid–water), with sorption being a key driver of contaminant mobility in natural systems (e.g., Freundlich partition constants concerning contaminant affinity toward soils and clays) and removal in engineered systems [contaminant affinity toward biochars, carbon nanotubes (CNTs), and activated carbons].^32,33^ Several studies have reported sorption capacities (Freundlich/Langmuir) of soils,^34−42^ biochars,^43,44^ and activated carbon^15,45−47^ for neonicotinoids and select neonicotinoid transformation products. We recently reported on the extensive sorption of parent neonicotinoids and pharmacophore-altered imidacloprid transformation products to granular activated carbon (GAC, 60–150 mg g^–1^), powdered activated carbon (PAC, 50–90 mg g^–1^), and CNTs (30–140 mg g^–1^).^47^ Although neonicotinoids and their transformation products sorbed extensively to black carbon, sorption to GAC for the pharmacophore-altered transformation products desnitro-imidacloprid and imidacloprid urea was significantly lower than was observed for the parent, imidacloprid.^47^ Experiments with functionalized and nonfunctionalized carbon nanotubes (nF-CNTs) indicate that the pharmacophore is a significant driver in neonicotinoid sorption,^47^ similar to previous observations in soils.^37−39^ This phenomenon is of particular concern because the neonicotinoid pharmacophore (i.e., the nictroimine/cyanoimine functional group that primarily drives neonicotinoid insecticidal propensity) can be readily transformed in the environment both biotically^20−24^ and abiotically (e.g., photolysis,^25^ hydrolysis,^15,16,25,26^ and chlorination^16^).

Prediction models can help to estimate the fate of novel transformation products in natural and engineered systems when experimental methods are limited by the lack of commercially available analytical standards. A common approach to predicting contaminant fate in environmental chemistry is the use of poly parameter linear free energy relationships/linear solvation energy relationships (pp-LFERs/LSERs).^32,48−50^ The pp-LFER/LSER relates the free energy terms of a contaminant (solute/Abraham parameters) to the intermolecular interactions associated with two given phases (e.g., *K*_ow_, *K*_oc_; solute/system parameters include excess molar refractivity, polarizability/dipolarity, hydrogen bond donor/acceptor interactions, and McGowan volume).^33,48−60^ The large number (often *n* > 100) and diverse structures used to generate and verify pp-LFER/LSER models^32^ is often a strength of these models; however, this also causes these models to be overly generic and historically applicable to hydrophobic/nonionized legacy contaminants rather than polar/ionizable/highly targeted emerging contaminants such as neonicotinoids.^32^ Utility of pp-LFERs/LSERs is further limited with more complex, heterogenous, phases (e.g., biochar, CNTs, and GAC).^47^ Researchers have begun using machine learning and neural networks to find quantitative structure–activity relationships (QSARs) to describe carbon surfaces and adjust pp-LFERs/LSERs to better predict sorption;^61−69^ however, these models may continue to lead to inaccurate predictions for contaminants like neonicotinoids where sorption is driven by physiochemical interactions with the pharmacophore.

The objective of this study was to combine computationally derived QSAR parameters for neonicotinoids with experimentally determined Freundlich partition constants (log *K*_F_ for CNTs and GAC) to accurately model neonicotinoid/transformation product sorption, providing the ability to accurately predict the fate of novel transformation products in drinking water treatment plants. Our approach was to use a representative group of contaminants (i.e., neonicotinoids) as a proof-of-concept in developing a method capable of accurately reflecting the fate of contaminants with unique, highly specified, structure–function moieties in a complex system like GAC—not necessarily to vet a specific model solely for neonicotinoids. Using a tailored model wherein QSAR descriptors were selected that best reflect neonicotinoid interactions in carbon–water systems, we were able to more accurately predict neonicotinoid/transformation product partitioning compared to traditional methods and predict partitioning for novel transformation products where experimental work is difficult because there are no commercial standards currently available for such compounds.

## Methods

### Experimental Data

Sorbents used in this study were identical to those reported in our prior work:^47^ F200 Calgon GAC (F200 GAC), nF-CNTs (190 m^2^ g^–1^), carboxylic acid functionalized/oxidized CNTs (O-CNTs, 120 m^2^ g^–1^), and amine-functionalized CNTs (N-CNTs, 220 m^2^ g^–1^).^47^ Further information regarding sorbents is provided in the Supporting Information.^70^ Sorption data for imidacloprid, clothianidin, thiacloprid, thiamethoxam, imidacloprid urea, and desnitro-imidacloprid were analyzed from our previously published data.^47^ Additional batch sorption isotherms for acetamiprid, dinotefuran, and thiacloprid amide were generated for this study and quantified following our previously reported methods (see the Supporting Information for details, Figures S1–S3, and Tables S1 and S2).^47^ All experiments were conducted at pH 7 in buffered solutions, as we had conducted previously,^47^ where neonicotinoids are in their neutral/nonionic state (Table S3) and hydrolysis will not be relevant.^16^ The Freundlich model (eq S1) was used to fit isotherms in this study (vs the Langmuir model used in our previous study) because it encompasses a wide range of sorption mechanisms and is the sorption parameter most commonly used in studies modeling contaminant partitioning to black carbon.^61−68^ Further details regarding chemicals, sorbents, and isotherms are provided in the Supporting Information along with Freundlich sorption parameters (Table S1). Freundlich partition constants used in model development and testing were log-transformed in accordance with current pp-LFER standards (Table S2).^32,54^

### QSAR Descriptors

QSARs were generated with the Spartan’18 Parallel Suite Quantum Mechanics Program (×86/Darwin), release 1.4.4 (Oct 9 2019) Wavefunction, Inc. and are described fully in the Supporting Information (Table S4). QSAR parameters were determined based on each compound’s ground state equilibrium geometry in water (we note that these computations can be impacted by the redox conditions, which can introduce some uncertainty). Computations were performed using a ωB97X-D/6-31G* (method/basis set) and restricted hybrid model (SCF model HF-DFT using Pulay DIIS + Geometric direct minimization, polarizable continuum solvation model). Graphics of neonicotinoids (presented in the Supporting Information) were created using the Spartan’18 Parallel Suite graphics program at high resolution (volume = density and volume = electron ionization potential). A total of 32 descriptors were obtained directly from Spartan that describe the electrical, quantum, and geometric molecular properties of a given compound. An additional 12 descriptors were calculated based on the molecular formula that describe constitutional molecular properties of a given compound (e.g., number of C, H, N, O, S, and double bond equivalence, Table S4).^56^ QSAR descriptors are fully described in the Supporting Information.

### Databases, Tools, and Data Mining

Experimentally determined Abraham solute parameters and literature log *K*_ow_ values for a set of 76 pesticides/pharmaceuticals were obtained from Tülp et al.^53^ for model development and comparisons (Table S5). Log *K*_ow_ values for the set of 76 pesticides/pharmaceutical and neonicotinoids were used in the initial model development to compare model approaches and quantify model performance (i.e., predicted vs measured results), as is commonly conducted for the initial evaluation^32,54^ (see Supporting Information for details). Predicted Abraham solute parameters for neonicotinoids were calculated based on SMILES structures^71^ (Table S6) using the UFZ-LSER database^72^ and subsequently used to determine pp-LFER-predicted log *K*_ow_ and log *K*_F_ (see Supporting Information).

### Model Development and Validation

Multiple linear regression (MLR) models were optimized using five QSAR parameters (the same number of β-parameters as the pp-LFER/LSER; eq 1) to model neonicotinoid/transformation product partitioning in octanol–water (based on literature log *K*_ow_)^6^ and CNT–water systems (based on experimental log *K*_F_).
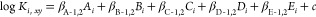
1where β-parameters A–E represent different QSAR parameters chosen to describe a given, two-phase, partition system (i.e., phase 1 and phase 2), *c* is the intercept for the given partition system, and the capital letters represent the corresponding QSAR parameters for a given contaminant. We initially chose to develop five-parameter models to be consistent with the models most currently used (i.e., the pp-LFER that uses the following five solute/system parameters: (*E* [molar refractivity], *S* [dipolarity/polarizability], *A* [hydrogen bond donor/electron acceptor], and *B* [hydrogen bond acceptor/electron donor]).^32,72^ Additionally, GAC is a complex, heterogenous surface that we previously identified as undergoing numerous interactions that contribute to neonicotinoid sorption (e.g., electrostatic interactions with the pharmacophore, π–π stacking, hydrogen bonding, and nonspecific interactions).^47^ Therefore, multiple parameters are required to discern the subtle structural nuances with significant impacts on the sorption propensity of neonicotinoids/transformation products. Initial models for log *K*_ow_ were constructed around a set of 76 pesticides/pharmaceuticals^53^ to compare two modeling approaches, which are presented in detail in the Supporting Information: (A) a traditional approach (akin to the pp-LFER with Abraham-inspired solute/system parameters) that served as a status quo benchmark for predicting partitioning and (B) a tailored approach that is composed of QSAR descriptors most correlated to the specific data set and partition system.

#### Traditional Approach (Approach A)

Because only one neonicotinoid (clothianidin) had available solute parameters in the UFZ-LSER database that were experimentally determined, a pp-LFER-inspired QSAR model was generated by iteratively combining the QSAR parameters most significantly correlated (i.e., Pearson r correlation with a *p*-value <0.2) to experimentally determine Abraham solute descriptors. This was conducted as a means to compare our tailored QSAR model predictions to those that would be generated with a more traditional pp-LFER. Experimentally determined solute parameters were iteratively combined with the Spartan parameters for the set of 76 pesticides/pharmaceuticals^53^ (*E*, *S*, *A*, *B*; Tables S10 and S11) until an optimal model describing octanol–water partitioning was obtained. The V parameter (McGowan volume) was calculated and not substituted with a Spartan QSAR descriptor (eq S2).^32,73^

The traditional pp-LFER (approach A) did not accurately predict neonicotinoid partitioning in relatively simple systems like octanol–water (log *K*_ow,_ used here to check model performance in relation to the pp-LFER). Clothianidin log *K*_ow_ (the only neonicotinoid with experimentally determined solute parameters)^53^ was overpredicted by 0.99 log-units (1.69 vs literature value of 0.70). Neonicotinoid log *K*_ow_ (with 95% confidence intervals) was overpredicted by 0.4–1.3 log units (Figure S4, QSAR values in Table S12), where neonicotinoids with lower log *K*_ow_ values being the most overpredicted.

#### Tailored Approach (Approach B)

Alternatively, the tailored approach was generated by iteratively combining five QSAR parameters that were the most significantly correlated/relevant with log *K*_ow_ partitioning (*p* < 0.5) for the 76 pesticides/pharmaceuticals^53^ and neonicotinoids independently and compared to assess model performance. Models for neonicotinoid CNT partitioning (log *K*_F_) were generated using experimentally determined *K*_F_ values for neonicotinoid sorption to nf-CNTs and O-CNTs (CNTs that best reflect the GAC surface).

MLR models were iteratively constructed and then analyzed using training (for model generation) and testing (model validation) sets of compounds in accordance with OECD requirements for proper development of QSAR models.^74^ For comparisons betwixt the traditional and tailored approaches (approaches A and B) involving the set of 76 pesticides/pharmaceuticals, the training set included all 76 pesticides/pharmaceuticals (except in the event that a QSAR parameter could not be calculated for a given compound), while the testing set (i.e., external validation) was composed of the 6 neonicotinoids (imidacloprid, clothianidin, thiamethoxam, thiacloprid, acetamiprid, and dinotefuran) and 3 neonicotinoid transformation products (imidacloprid urea, desnitro-imidacloprid, and thiacloprid amide) that are used throughout the study. For MLRs generated solely around neonicotinoids, the training/testing set was split 80/20 (as is most commonly conducted),^61,64,65,67,68^ with imidacloprid, clothianidin, thiacloprid, dinotefuran, imidacloprid urea, and desnitro-imidacloprid as the training set and thiamethoxam, acetamiprid, and thiacloprid amide as the testing set (for external validation). Although it is preferable for testing/training sets to be assigned randomly, they were specifically chosen in this study due to the small sample size available and known importance of the various neonicotinoid structural elements on sorption propensity. Thus, thiamethoxam (structurally hindered nitro-pharmacophore), acetamiprid (cyano-neonicotinoid pharmacophore), and thiacloprid amide (neonicotinoid transformation product) were selected to test model performance toward a set of structurally diverse neonicotinoids.

Models were internally validated statistically and chosen based on highest *R*^2^, *R*_adj_^2^, lowest RMSE, and the *F*-value/corresponding *p*-value of the overall model (*p* < 0.05). Each model parameter was required to have a significance of *p* < 0.1 (unless otherwise stated, see Supporting Information). A *p*-value of 0.1 was used rather than 0.05 due to the small sampling size. The cutoff for the variation inflation factors (VIFs), which account for multicollinearity between model parameters, was <10 to ensure that there was minimal redundancy between parameters within a given model.^75,76^ Models were deemed predictive if the 95% confidence interval of the predicted log *K*_ow_ or log *K*_F_ value overlapped with the literature (log *K*_ow_) or experimentally determined (log *K*_F_) values and the predicted constant was within 0.5 log-units of the literature or experimental value.

Using the tailored model (approach B), with a suite of pesticides/pharmaceuticals, neonicotinoid log *K*_ow_ continued to be overpredicted (by 0.5–2.0 log-units, Figure S4, see Supporting Information for details). As described earlier, comparing predicted and reported *K*_ow_ is commonly used for model evaluation.^32,54^ Through an iterative process testing dozens of models, we generated a tailored neonicotinoid log *K*_ow_ model (Table S15 and Figure S6) with an RMSE of 0.01723 that could accurately predict acetamiprid and thiacloprid amide log *K*_ow_ during external validation (Table S13C and Figure S4E). Multiple QSAR parameters for the neonicotinoid log *K*_ow_ model relate to energy terms (e.g., minimum local ionization potential, total energy, and polarizability), each of which represents a different electrostatic property with distinct sorption mechanisms (e.g., ability to ionize/form a dipole/undergo electron donor/acceptor interactions). Many of these specific interactions are largely overlooked in both the traditional pp-LFER, particularly when using a suite of structurally dissimilar contaminants.

### Predictions for Novel Transformation Products

MLRs developed and validated for neonicotinoids were subsequently used to predict the log *K*_ow_ and log *K*_F_ of neonicotinoid transformation products (some of which lack commercially available standards): imidacloprid olefin, 5-hydroxy imidacloprid, clothianidin methyl urea, and eight novel chlorination and hydrolysis products we previously identified via high-resolution mass spectrometry.^16^ Each predicted partition constant is reported with the 95% confidence range.

### Analysis, Quality Assurance, and Statistics

Information regarding sample quantification via liquid chromatography tandem mass spectrometry was previously reported and is provided in the Supporting Information and Tables S8 and S9. Experimental QA/QC was previously reported and is also provided in the Supporting Information. All model fitting and statistical analyses were conductedwith Graphpad Prism 9 software (La Jolla, CA) at a 95% confidence level unless otherwise stated.

## Results and Discussion

### Modeling Neonicotinoid Sorption to CNTs and GAC

**Figure 1:**
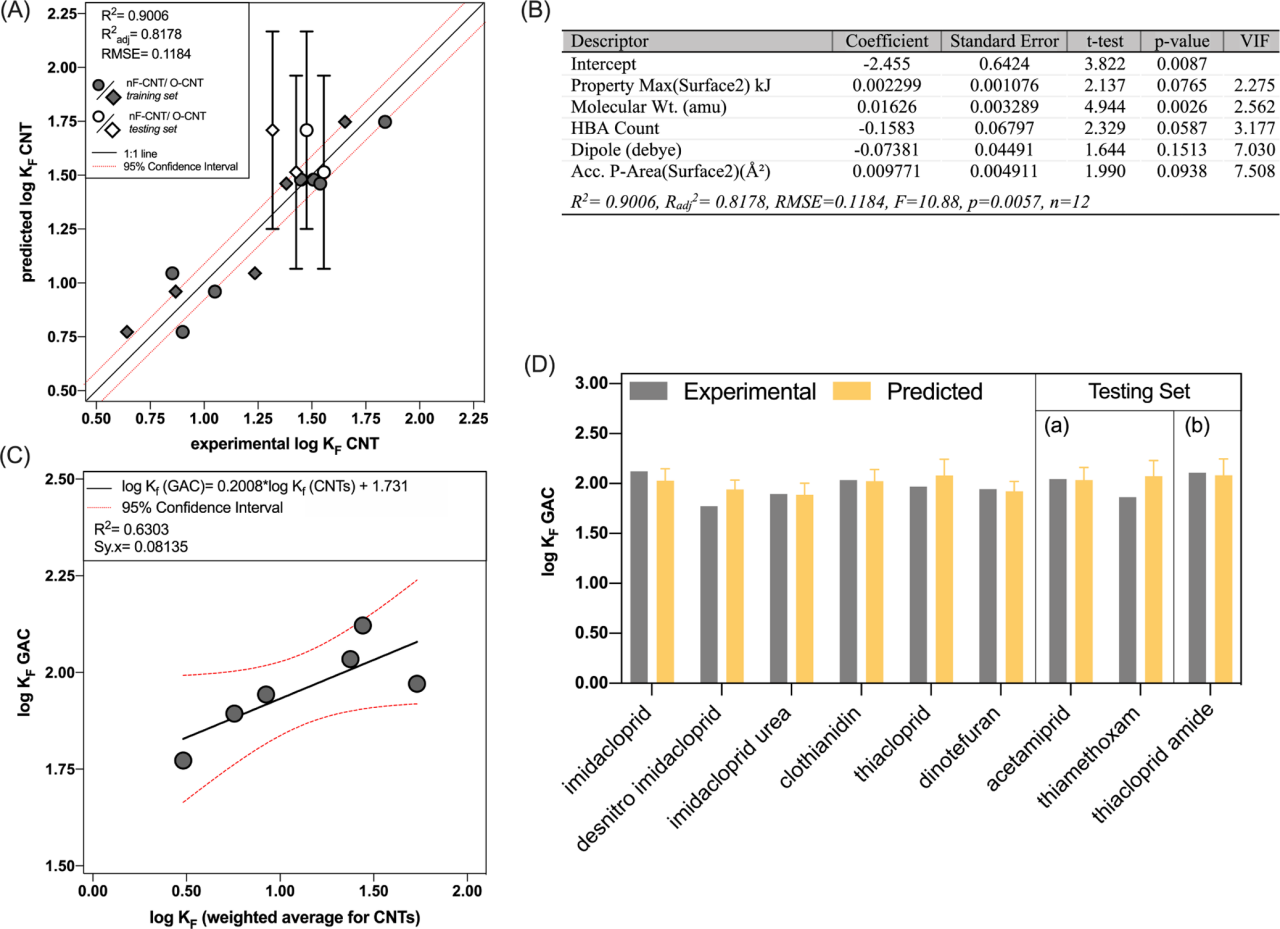
(A) MLR for predicting neonicotinoid and transformation product log *K*_F_ for nF- (circles) and O-CNTs(diamonds). Gray shapes indicate neonicotinoids used in the training set, while white shapes are those in the testing set. Thiacloprid amide is not pictured because no sorption experiments with CNTs were conducted with thiacloprid amide, only GAC sorption was measured for thiacloprid amide (as a fully external validation). (B) Table of the optimal QSAR descriptors (and corresponding coefficients, significance, and multicollinearity [VIF]) for modeling neonicotinoid sorption to nF-/O-CNT (log *K*_F_). This model excludes acetamiprid, thiamethoxam, and thiacloprid amide, which were used in external validation. (C) Simple linear regression relating the weighted average log *K*_F_ value for nF-/O-/N-CNTs to the log *K*_F_ determined with F200 GAC. The weighting used was 1.75*nF-CNT, 1.25*O-CNT, and 1*N-CNT. (D) Experimental GAC log *K*_F_ for each neonicotinoid and transformation product (gray) compared to the predicted GAC log *K*_F_. Within the testing set, there are two groups: group (a) consisting of acetamiprid and thiamethoxam where external validation was conducted in the CNT MLR and GAC extrapolation and group (b) consisting of thiacloprid amide where fully external validation was only conducted following GAC extrapolation.

We recently reported^47^ that when the neonicotinoid nitroimine pharmacophore is transformed to an imine/amine group (i.e., imidacloprid forming desnitro- and urea metabolites), the sorption propensity of the products to CNTs and GAC significantly decreases, which is thought to be driven by alterations to the electrostatic and/or hydrogen bonding interactions that occur between the neonicotinoid pharmacophore and the carbon surface. Over a hundred unique models were generated that describe neonicotinoid/transformation product sorption (log *K*_F_) to nF-CNTs and O-CNTs (parameter correlations in Table S17). The optimal QSAR model (RMSE = 0.1184) generated to describe neonicotinoid sorption to CNTs was a five-parameter model based on maximum surface energy (kJ), molecular weight (amu), hydrogen bond acceptor count, dipole moment (debye), and accessible polar surface area (Å^2^), with each parameter statistically significant in accordance with OECD guidelines (Figure 1A,B).^77^ This model was externally validated using thiamethoxam and acetamiprid, which were predicted within 95% confidence of the experimentally determined nF-/O-CNT log *K*_F_ values. Thiamethoxam log *K*_F_ was overpredicted by 0.4 log-units, while acetamiprid was overpredicted by 0.2 log-units (Figure 1A). A neonicotinoid product, thiacloprid amide, was completely excluded from both the CNT model training and testing sets and served to externally validate subsequent CNT-to-GAC log *K*_F_ extrapolation.

Based on the most important interactions for neonicotinoid sorption to black carbon,^70^ we used nF-CNTs (primarily graphitic surface) and O-CNTs (oxygen-containing surface sites) to generate a MLR model representing the main neonicotinoid interactions with GAC (i.e., highly graphitic with oxidized surface groups).^78^ A simple linear regression extrapolated neonicotinoid CNT sorption to more complex, and environmentally relevant, F200 GAC sorption. The log *K*_F_ of neonicotinoids/transformation products for the three CNTs (nonfunctionalized, oxidized, and amine) was weighted to best reflect the relative surface characteristics of the F200 GAC in our prior study^70^ (Figure 1C). A weighting ratio of 1.75*nF-CNT, 1.25*O-CNT, and 1*N-CNT was used because F200 GAC has been previously characterized by researchers as ∼80% carbon, ∼10% oxygen, and <2% nitrogen; and considering that nF-CNTs were >98% carbon, O-CNTs were ∼5% oxygen, and N-CNTs were ∼6% nitrogen (according to vendor specifications).^78^ Weighting was used in the extrapolation to GAC but not the CNT-MLR in order to include/reflect how the changes to carbon surface groups impact neonicotinoid sorption. N-CNTs were not included in the CNT MLR to avoid overly biasing the CNT regression with a surface property that represents little of the GAC surface.^78^ CNT-to-GAC extrapolation provided a reasonable regression (Figure 1C) with accurate predictions for sorption to F200 GAC for thiamethoxam (0.21 log-units overpredicted), acetamiprid (0.01 log-units underpredicted), and thiacloprid amide (0.03 log-units overpredicted, Figure 1D). Of particular note is the ability of this approach (i.e., CNT MLR and extrapolation to GAC) to accurately predict the sorption of thiacloprid amide to F200 GAC (predicted within 0.03 log-units), which was completely excluded from the CNT model development/verification as a form of fully external validation.

**Figure 2:**
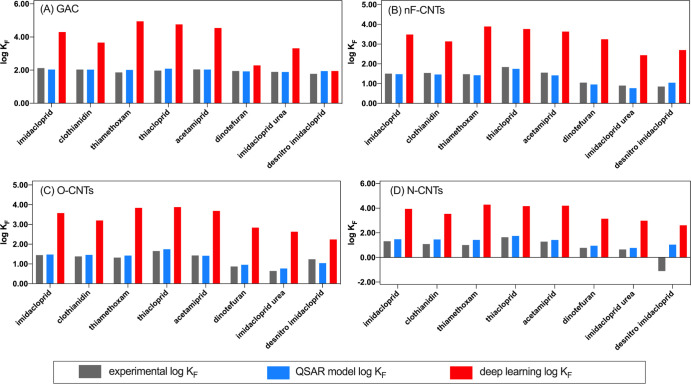
Experimental log *K*_F_ (gray) vs predicted log *K*_F_ using the neonicotinoid-tailored QSAR sorption model (blue) and the deep learning model presented by Sigmund et al.^61^ in a Matlab interface (red). Deep learning predictions were conducted for (A) Calgon F200 GAC, (B) nF-CNTs, (C) O-CNTs, and (D) N-CNTs.

A growing body of research is employing machine learning and/or neural networks to use surface properties of black carbon (e.g., surface area, C/H, and percent oxygen) to improve pp-LFER/LSER predictions for contaminant sorption to activated carbon, CNTs, and biochars.^61−68,75^ We entered information regarding the surface properties of our sorbents (nF-/O-/N-CNTs, F200 GAC; see Supporting Information) and system pH (Table S18),^79^ and the SMILES-predicted Abraham solute parameters (Table S6) into a recently published model based on deep learning^61^ to predict neonicotinoid sorption (log *K*_F_) for each sorbent studied. With the deep learning model, neonicotinoid log *K*_F_ for CNTs and GAC were 1.0–3.0 log units higher than those determined experimentally or predicted with our QSAR-based MLR models (Figure 2); this is likely due to the use of an overly general pp-LFER. Similarly, the deep learning model was unable to discern/predict the relative rank-order of neonicotinoid sorption (Table S19), highlighting how the pp-LFER approach is insufficient for neonicotinoids that have a specific pharmacophore-driven sorption mechanism. Thus, the use of QSAR parameters targeting neonicotinoids specifically highlights the most important interactions that drive neonicotinoid sorption—particularly energy terms that are overlooked/underweighted when using the pp-LFER and/or contaminants with little structural similarities to the contaminant of interest. These discoveries may improve prediction for other compound classes where key portions of a molecule are instrumental to partitioning; for example, transformation of fipronil (i.e., sulfonyl group to sulfide/sulfone products)^80,81^ or phenoxyalkanoic herbicides (i.e., to phenolic products)^82^ results in increased sorption to soil.

### Predicting Novel Neonicotinoid Transformation Product Partitioning

Alterations to the neonicotinoid structure are known^70^ and are here predicted to slightly alter neonicotinoid octanol–water partitioning and sorption to black carbon. The neonicotinoid log *K*_ow_ and log *K*_F_ CNT-MLR models (CNT-to-GAC extrapolation) (Figure 1) were used to predict partitioning of multiple neonicotinoid transformation products formed via microbial degradation, photolysis, chlorination, and hydrolysis (Table 1)^15,16,20−26,83^ for which no experimental data are currently available due to the lack of commercially available standards.

**Table 1:**
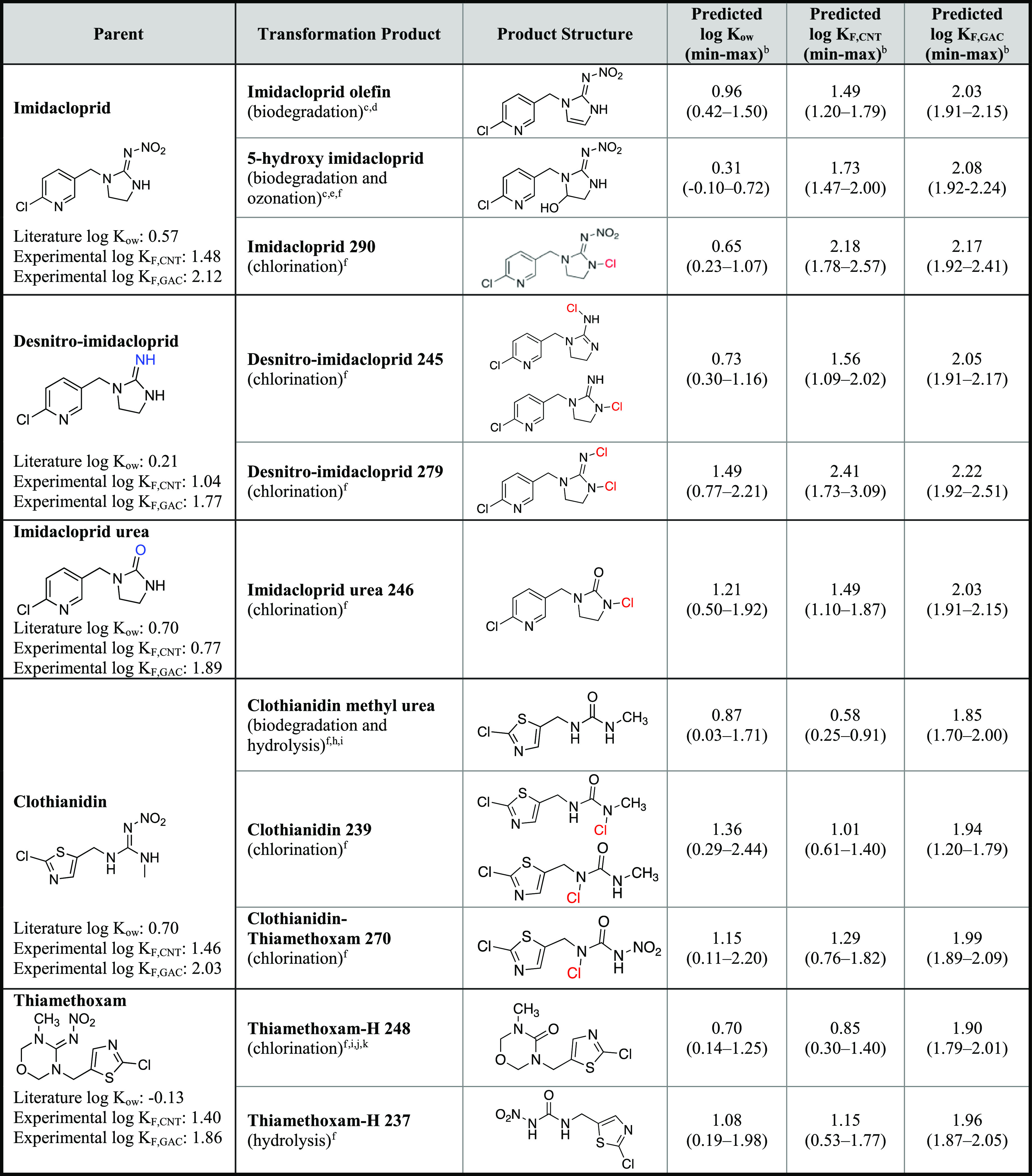
Predicted Partition Coefficients (*K*_ow_) and Constants (*K*_F_) for Neonicotinoid Transformation Products and Transformation Products^a^

a Predictions are presented with 95% confidence intervals (min–max) outlining the range in predicted Freundlich partition constants.

b 95% confidence interval.

c Dai et al.^20^

d Lu et al.^21^

e Bourgin et al.^83^

f Klarich Wong et al.^16^

g Mulligan et al.^22^

h Mori et al.^23^

i Todey et al.^25^

j Liqing et al.^26^

k Pandey et al.^24^

#### Microbial Degradation and Photolysis Products

**Figure 3:**
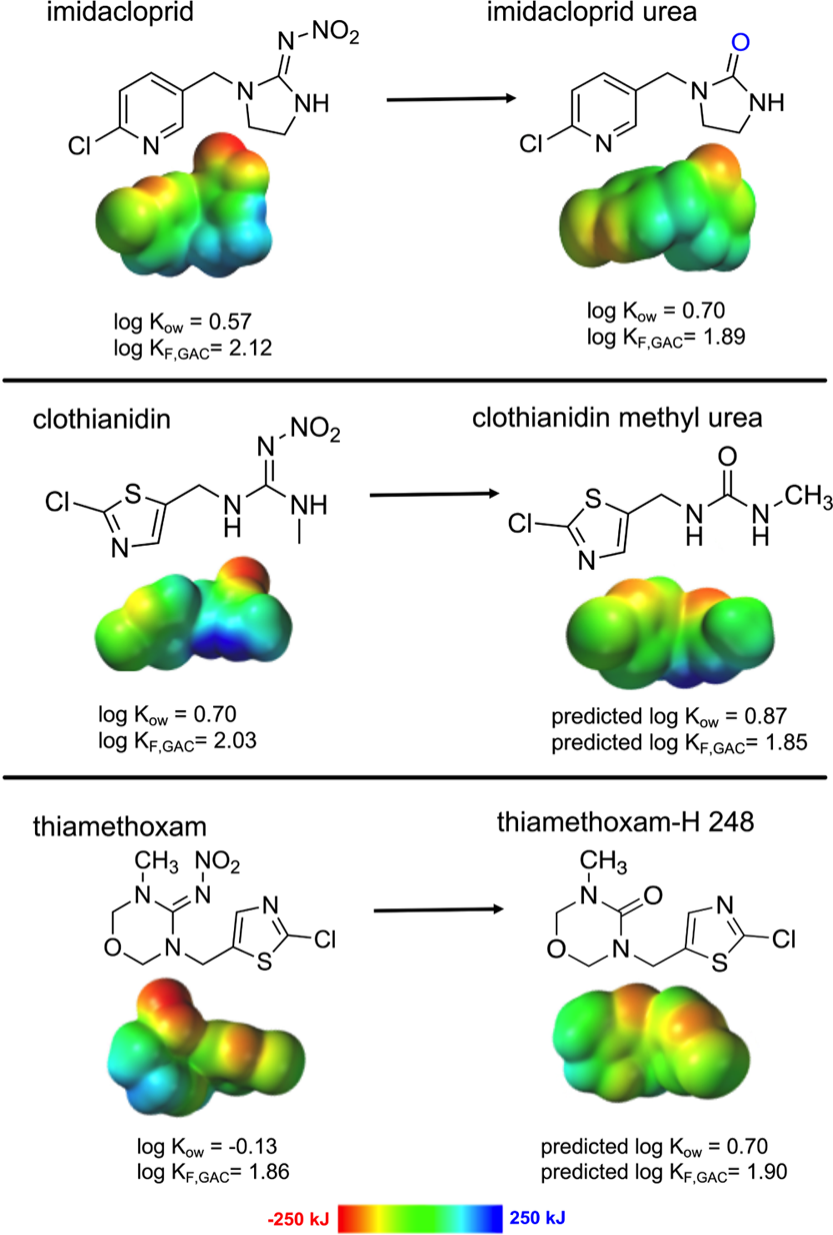
Electron ionization energy diagrams for imidacloprid, clothianidin, and thiamethoxam and their corresponding urea transformation products. Arrows show parent to product relationships. Electron ionization energy diagrams range from −250 (red) to 250 (blue) kJ and plot with an iso val of 99.8% 0.0006 e^–^/au^3^.

The microbial transformation product 5-hydroxy imidacloprid (hydroxy addition) is predicted to have a slightly lower log *K*_ow_ and log *K*_F,GAC_ and higher log *K*_F,CNT_ compared to parent imidacloprid (Figure S7). Alternatively, microbial transformation of imidacloprid into imidacloprid olefin is predicted to increase log *K*_ow_ and decrease log *K*_F,GAC_ (with no impact on log *K*_F,CNT_, Figure S7). Through our previous isotherm analyses,^70^ transformation of imidacloprid into imidacloprid urea (loss of the nitroimine) results in diminished sorption to CNTs and GAC (log *K*_F_) and here is predicted to slightly increase log *K*_ow_ (Figure 3).^6^ Similarly, clothianidin methyl urea, which is also formed through the loss of the nitroimine group, is predicted to slightly increase log *K*_ow_ and yield a large decrease in log *K*_F_ for CNTs and GAC (Figure 3). These predictions indicate that upon addition of polar groups (e.g., −OH in 5-hydroxy imidacloprid), there is a slight decrease in log *K*_ow_ and log *K*_F_ (CNT and GAC), while upon loss of the polar nitroimine (e.g., nitroimine in imidacloprid urea and clothianidin methyl urea), there is a predicted increase in log *K*_ow_ but decreased log *K*_F_. Although these predictions were often within 0.5 log-units of the parent partition coefficients, these predictions are consistent with the expected effects of polar groups on log *K*_ow_.^32^

#### Hydrolysis Products

The hydrolysis of thiamethoxam to THX-H 248 (i.e., loss of nitroimine) is predicted to follow the same trend as the other nitroiminine-to-urea transformation products, with increased log *K*_ow_ and lower CNT and GAC log *K*_F_ compared to parent thiamethoxam (Figure 3). For the hydrolysis product THX-H 237, log *K*_ow_ is predicted to increase greater than those for thiamethoxam and THX-H 248. THX-H 237 was also predicted to have a lower CNT and GAC log *K*_F_ compared to thiamethoxam, similar to what was predicted for the other hydrolysis product THX-H 248 (Figure S8). Thus, hydrolysis of thiamethoxam is expected to increase log *K*_ow_ and decrease CNT log *K*_F_.

#### Chlorination Products

**Figure 4:**
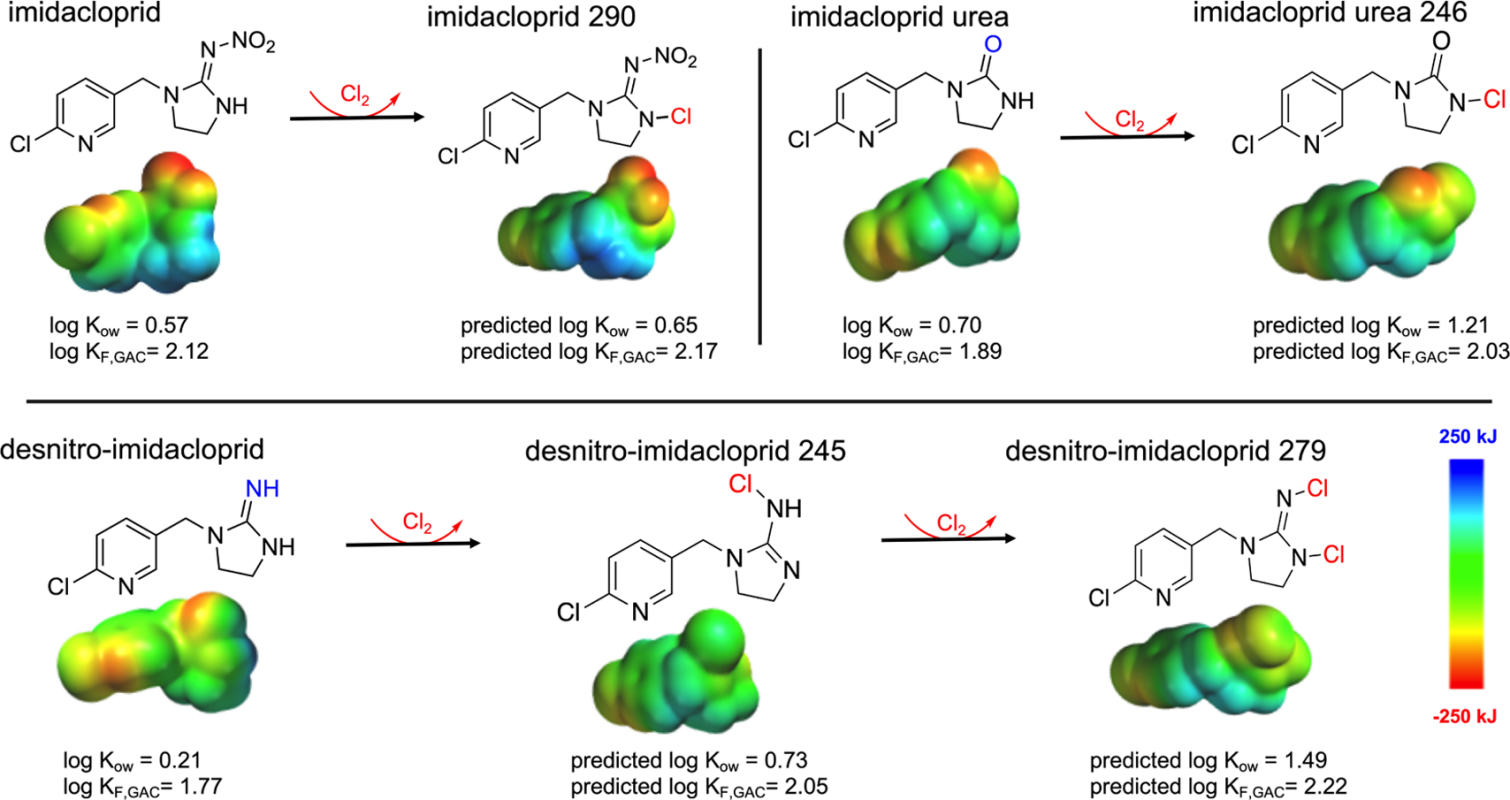
Electron ionization energy diagrams of imidacloprid, imidacloprid urea, and desnitro-imidacloprid with their corresponding chlorination products. Electron ionization energy diagrams range from −250 (red) to 250 (blue) kJ and plot with an iso val of 99.8% 0.0006 e^–^/au^3^.

All the chlorinated products we proposed in prior work by Klarich Wong et al.^16^ are predicted to have a higher log *K*_ow_ than their respective precursor (by 0.1–0.8 log-units), which is consistent with what is known regarding the impacts of the degree of chlorination and hydrophobicity (Table 1).^32,84^ This trend is most illustrative with imidacloprid, desnitro-imidacloprid, and imidacloprid urea, which were all predicted to have a higher log *K*_F_ for CNTs and GAC following degree of chlorination (e.g., the log *K*_ow_ and log *K*_F_) for the di-chlorinated desnitro-imidacloprid [desnitro-imidacloprid 279] > mono-chlorinated desnitro-imidacloprid [desnitro-imidacloprid 245] > desnitro imidacloprid, Figure 4). For clothianidin, the chlorinated transformation products CLO 239 and CLO-THX-H 270 are most structurally similar to clothianidin methyl urea (Figure S9). The chlorinated version of clothianidin methyl urea (CLO 239) had a greater predicted log *K*_ow_ and log *K*_F_ compared to the methyl urea transformation product, analogous to predictions for imidacloprid urea. The product CLO-THX-H 270 is predicted to have a lower log *K*_ow_ and higher log *K*_F_ than the product CLO 270, consistent with what is known regarding the impact of polar groups (e.g., nitro) on log *K*_ow_ and log *K*_F_ as observed with imidacloprid and its transformation products.^6,70^

### Model Discussion

The goal of this study was to provide a proof-of-concept development of a more appropriate model for predicting contaminant sorption using relatively attainable inputs (i.e., computational and experimental data) translatable to environmentally relevant surfaces (e.g., CNTs and GAC). The pp-LFER is the current standard for predicting contaminant partitioning, which is why we based our initial models on the pp-LFER and included five model parameters. Although we are cognizant of potential for overfitting, five parameters can at times be necessary for predicting contaminant fate in complex systems.^74,77^ For instance, we previously reported that neonicotinoid sorption to activated carbon likely involves hydrogen bonding, electron donor/acceptor interaction, π–π stacking, and pore diffusion.^70^ We also evaluated models with fewer parameters (see Supporting Information for details); however, only two model permutations (a two-parameter and three-parameter model) were capable of accurately predicting neonicotinoid/transformation product sorption, while satisfying OECD requirements with one exception (Figures S10, S11 and Table S20). Nevertheless, the two- and three-parameter models were not capable of predicting the impact of chlorination on desnitro-imidacloprid transformation products in a manner consistent with literature on the known effects of chlorination.^32,84^ The limits of the two- and three-parameter models with respect to desnitro-imidacloprid/transformation products are likely due to the inability of the fewer parameters to capture the impact of transformation on sorption. Thus, in the case of neonicotinoid/transformation product sorption, all five parameters are significant and likely necessary.^74,77^

## Conclusions

Although highly water soluble, neonicotinoids are capable of sorption to natural (soils and clay)^34−42^ and engineered sorbents (biochar, carbon nanotubes, and activated carbon).^43,44,46,70^ The extent to which neonicotinoids and transformation products sorb is highly dependent on the electrostatic properties of the neonicotinoid pharmacophore,^37,39,70^ as well as sorbent properties like organic carbon content (soil, Figure S12)^34−42^ and aromaticity (biochar),^43,44^ making these results more broadly applicable to the environment beyond carbon nanotubes and activated carbon. We demonstrate here that combining neonicotinoid SARs with experimental data can more accurately predict sorption than current approaches and further highlights the altered sorption interactions between neonicotinoids and their transformation products. Additionally, the use of predictive models is of particular utility when analytical standards for transformation products are not commercially available. Because the neonicotinoid structure is critical not only to its selectivity/toxicity^28,29,31,85^ but also to its fate in natural and engineered systems (sorption, chlorination, and hydrolysis),^16,25,70^ there is a growing need to accurately predict the fate of novel transformation products a priori in order to prioritize research (e.g., use of accurate log *K*_ow_ values to predict human toxicity via bioaccumulation or ability to cross the blood–brain barrier).^86−89^

We demonstrate that the current approach to predicting contaminant fate in the environment (i.e., pp-LFERs/LSERs) falls short for accurately predicting neonicotinoid partitioning in simple (log *K*_ow_) and complex systems (log *K*_F_ carbon nanotubes and GAC). This could be due to the highly polar nature of neonicotinoids and electrostatically driven SAR associated with the pharmacophore, which is not well represented by the compounds predominately included in available pp-LFERs. By tailoring our QSAR models to neonicotinoids and transformation products specifically, we were able to generate a model that could more accurately predict neonicotinoid fate with a limited amount of lab work and extrapolate to a much more complex system (e.g., GAC). Although the models presented herein are focused on a limited group of contaminants (i.e., neonicotinoids), our relatively simple and targeted approach could have significant utility for more accurately predicting the fate of other groups of emerging contaminants in a variety of environmental systems and highlights important interactions that drive sorption of polar compounds (e.g., energy terms). Analogous models could be constructed for the growing number of target-specific contaminants where the structural integrity of the pharmacophore is crucial to toxicity and/or environmental partitioning (e.g., fipronil), or for contaminants classes where key structural characteristics are conserved but vary between compounds (e.g., PFASs).

Depending on the contaminants and complexity of the system in question, more or fewer parameters could be used to model partitioning at the discretion of the analyst. Our goal was to employ a representative class of contaminants—known to be poorly represented with traditional models used to predict environmental fate—to demonstrate a model development approach that uses relatively simple computational and experimental inputs to more accurately predict and understand contaminant fate in natural and engineered systems. We also gain insights as to relevant processes based on the model development (e.g., energy terms). This approach (i.e., combining limited laboratory data with QSAR) could lower experimental burdens and be used as a preliminary assessment of the fate and potential impact of other novel neonicotinoid transformation products, or aid in novel transformation product or next-generation compound risk assessment.

## Supporting Information Available

Additional method details, quality assurance/control, and additional detailed data/results/analysis (PDF)
