# Supplementary material for: Combining Experimental Sorption Parameters with QSAR to Predict Neonicotinoid and Transformation Product Sorption to Carbon Nanotubes and Granular Activated Carbon
Source: ACS ES T Water. 2022 Jan 5;2(1):247–58. doi: 10.1021/acsestwater.1c00492 (PMC8762664; doi:10.1021/acsestwater.1c00492)
Supplement: Supplementary file 1 — ew1c00492_si_001.pdf [file ew1c00492_si_001.pdf]

Supporting Information

Combining Experimental Sorption Parameters with QSAR to Predict Neonicotinoid and Transformation Product Sorption to Carbon Nanotubes and Granular Activated Carbon

Danielle T. Webb,[§,¶] *Matthew R. Nagorzanski,*[§,¶] *David M. Cwiertny,*[§,¶,¥,€]

Gregory H. LeFevre[§,¶,*]

[§]Department of Civil & Environmental Engineering, University of Iowa, 4105 Seamans Center, Iowa City, IA 52242, United States; [¶]IIHR—Hydroscience & Engineering, 100 C. Maxwell Stanley Hydraulics Laboratory, Iowa City, IA 52242, United States; [¥]Center for Health Effects of Environmental Contamination, University of Iowa, 455 Van Allen Hall, Iowa City, Iowa 52242; [€]Public Policy Center, University of Iowa, 310 South Grand Ave, 209 South Quadrangle, Iowa City, IA 52242

***Corresponding Author:**
gregory-lefevre@uiowa.edu; Phone: 319-335-5655; 4105 Seamans Center for Engineering, University of Iowa, Iowa City IA, United States.

Supporting information contains: 34 pages total including supporting methods, supporting results, 12 figures, and 20 tables, and supporting references.

SUPPORTING METHODS

Chemicals. Neonicotinoids used in isotherm experiments were of a purity ≥95%: acetamiprid (Pestanal Fluka Analytical; 160430-64-8), dinotefuran (Pestanal Sigma Aldrich; 165252-70-0), thiamethoxam (99.5% Chem Service; 153719-23-4), thiacloprid amide (Pestanal Sigma Aldrich; 676228-91-4), and desnitro-imidacloprid hydrobromide (95% Chem Space; 105827-77-8). Isotopically labelled d_4-imidacloprid (Pestanal Sigma Aldrich; 1015855-75- 0) was used as an internal standard. Solvents used for chromatography (water, acetonitrile, and formic acid) were of Optima LCMS (liquid chromatography mass spectrometry) grade.

Sorbents. Commercially available sorbents granular activated carbon (Calgon F200 GAC, 670 m^2 g^{-1}) was used in our prior study to represent a material in use at a DWTP.[1] Similarly, multi-walled carbon nanotubes (CNTs) were described in our previous study.[1] Briefly, non-functionalized multi-walled carbon nanotubes (nF-CNTs, 190 m^2 g^{-1}), carboxylic acid/oxidized functionalized (O-CNTs, 120 m^2 g^{-1}), and amine-functionalized (N-CNTs, 220 m^2 g^{-1}) were used as a means to simplify neonicotinoid sorption to GAC by probing specific sorption mechanisms (i.e., π-π interactions, hydrogen bonding, and electron donor-acceptor interactions).[2] According to the vendor (cheaptubes.com) O-CNTs had a -COOH content of 4-5% and N-CNTs had a $-NH_2$ content of 5.5-8.5%. Although the vendor identified ranges of -COOH and $-NH_2$ surface functionalization, other surface groups may be present (e.g., N-CNTs can also contaim NH, $O{=}C{-}NH_2$, and C=N functionalities).

Isotherms. Sorption data for imidacloprid, clothianidin, thiacloprid, thiamethoxam, imidacloprid urea, and desnitro-imidacloprid were analyzed from previously published data.[1] Additional F-200 GAC isotherms were carried out for thiamethoxam and desnitro-imidacloprid to improve model fitting. Additional batch sorption isotherms for acetamiprid, dinotefuran, and thiacloprid amide were generated and quantified following our previously reported methods. F-200 GAC and CNT isotherms were generated for acetamiprid and dinotefuran while thiacloprid amide isotherms were only generated with F-200 GAC (**Figure S.1-S.2**). Isotherms for this study were fit with the Freundlich model (**Equation S.1**).

Equation S.1

$$q_e = K_F C_e^{1/n}$$

Where q_e is the equilibrium neonicotinoid adorbed concentration (mg g^{-1}), C_e is the equilibrium aqueous neonicotinoid concentration (mg L^{-1}), K_F is the Freundlich constant ([mg g^{-1}] [L mg^{-1}]$^{1/n}$), and n is the Freundlich exponent. Freundlich sorption parameters are provided in **Table S.1** with standard errors.

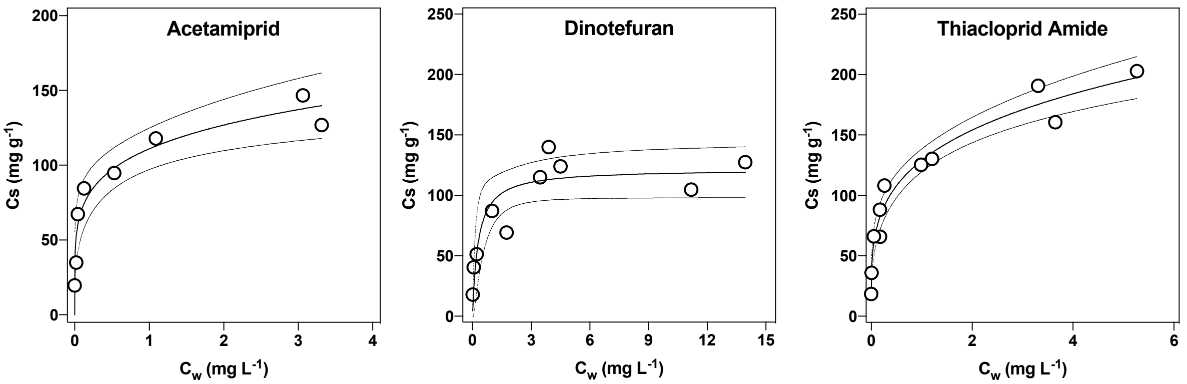

Figure S.1: Additional GAC sorption isotherms (not previously reported) for acetamiprid, dinotefuran, and thiacloprid amide fit with Freundlich model.

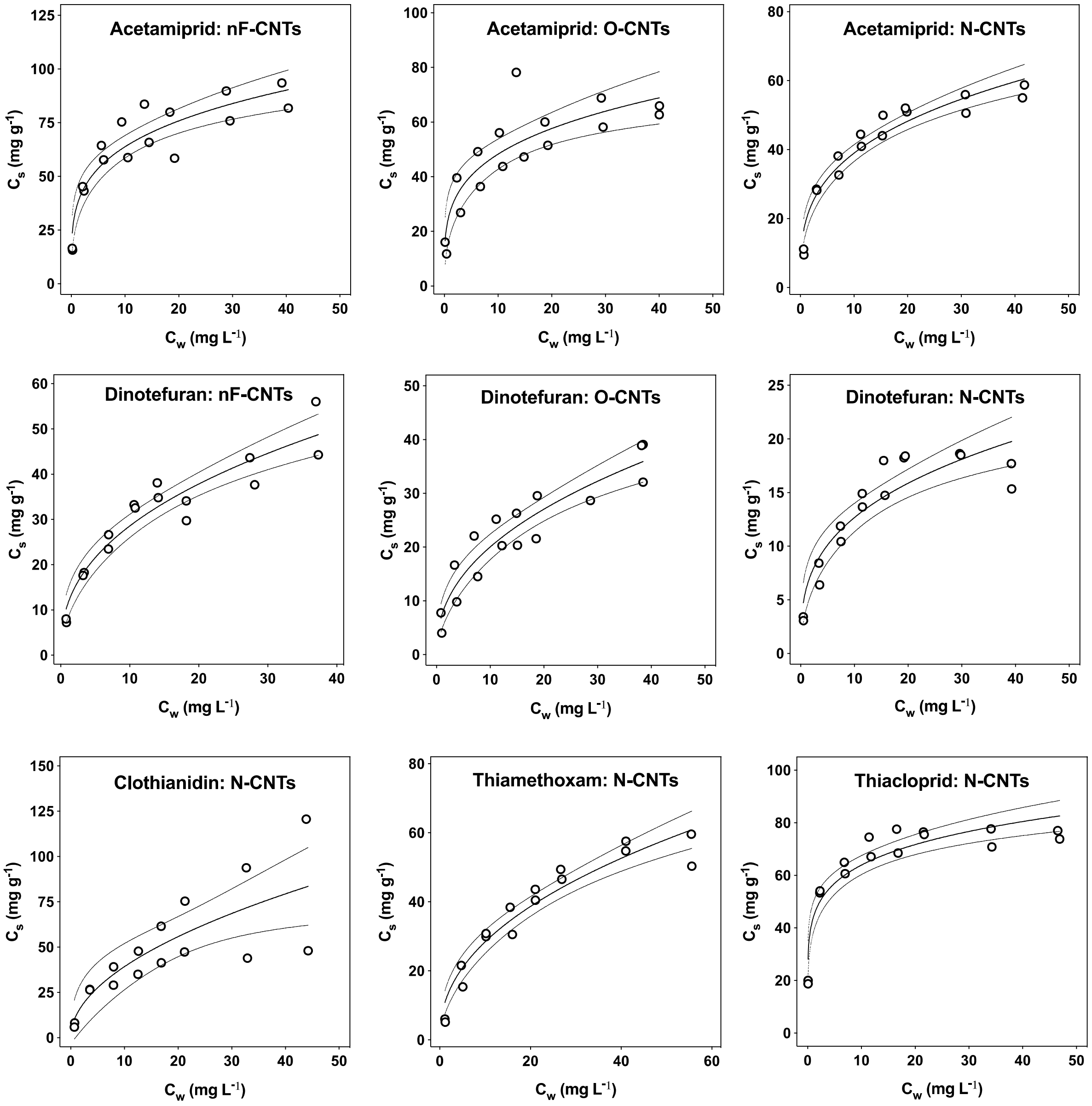

Figure S.2: nF/O/N CNT isotherms (not previously reported) for acetamiprid and dinotefuran and NH2-CNT isotherms (not previously reported) for clothianidin, thiamethoxam, and thiacloprid fit with the Freundlich model.

Table S.1: Freundlich isotherm coefficients for neonicotinoid sorption to nF-CNTs, O-CNTs, N-CNTs and F200 GAC.

	nF-CNTs (K_F ± SE)	nF-CNTs (n ± SE)	O-CNTs (K_F ± SE)	O-CNTs (n ± SE)	N-CNTs (K_F ± SE)	N-CNTs (n ± SE)	GAC (K_F ± SE)	GAC (n ± SE)
Imidacloprid	32.2 ± 4.3	3.03 ± 0.39	28.16 ± 3.47	3.86 ± 0.53	20.7 ±3.0	2.60 ±0.31	132 ± 21	ambiguous
desnitro imidacloprid	7.13 ± 2.3	3.26 ± 1.1	17.20 ± 1.90	3.21 ± 0.36	0.08 ±0.03	0.76 ± 0.06	59.2 ± 2.7	7.57 ± 0.97
Imidacloprid urea	7.95 ± 1.0	2.02 ± 0.16	4.39 ± 0.50	1.73 ± 0.11	4.39 ± 0.41	1.76 ± 0.09	78.3 ± 16	ambiguous
clothianidin	34.58 ± 3.5	3.85 ± 0.48	24.07 ± 3.10	3.42 ± 0.46	12.1 ± 5.5	1.96 ± 0.53	108 ± 18	ambiguous
thiamethoxam	29.87 ± 3.7	3.85 ± 0.57	20.79 ± 6.03	3.91 ± 1.3	10.2 ± 1.5	2.25 ± 0.22	73.0 ± 10	ambiguous
thiacloprid	69.07 ± 6.1	5.23 ± 0.80	45.06 ± 4.42	4.53 ± 0.67	43.6 ± 2.7	6.01 ± 0.75	93.5 ± 12	6.35 ± 2.9
Acetamiprid	35.93 ± 3.8	4.01 ± 0.57	26.72 ± 4.02	3.90 ± 0.77	19.2 ± 1.6	3.25 ± 0.30	111 ± 5.7	5.18 ± 0.93
Dinotefuran	11.21 ± 1.5	2.46 ± 0.27	7.38 ± 7.38	2.31 ± 0.29	6.0 ± 0.89	3.08 ± 0.46	87.7 ± 14	ambiguous
thiacloprid amide	N/A	N/A	N/A	N/A	N/A	N/A	129 ± 4.2	3.89 ± 0.34

Table S.2: Log partition coefficients for neonicotinoids.

	log Kow (CompTox)	nF-CNT log K_F	O-CNT log K_F	N-CNT log K_F	GAC log K_F
Imidacloprid	0.57	1.51	1.45	1.32	2.12
desnitro imidacloprid	0.205	0.85	1.24	-1.12	1.77
Imidacloprid urea	0.695	0.9	0.64	0.64	1.89
clothianidin	0.7	1.54	1.38	1.08	2.03
thiamethoxam	-0.13	1.48	1.32	1.01	1.86
thiacloprid	1.26	1.84	1.65	1.64	1.97
Acetamiprid	0.8	1.56	1.43	1.28	2.05
Dinotefuran	-0.644	1.05	0.87	0.78	1.94
thiacloprid amide	0.413	N/A	N/A	N/A	2.11

CNT Isotherm Method. CNT isotherms methodology was previously published and outlined briefly here.[1] CNTs (0.1 g) were added to deionized water (10 mL) in a glass scintillation vial and placed in a sonicator for five hours to promote CNT dispersion. Following dispersion, 0.1 mL of the CNT suspension was spiked into solutions of individual neonicotinoids (neonicotinoid concentrations spanned 5- 200 µM). Vials were then shaken and left in the dark to sit overnight (which was shown in our prior work to be an adequate amount of time to reach equilibrium for CNTs;[2] additionally, we have provided sorption kinetics data (**Figure S.3**) that demonstrates neonicotinoids rapidly achieve equilibrium). Once CNTs settled, a 1 mL aliquot was taken from the top of the solution for analysis by liquid chromatography tandem mass spectrometry. All CNT isotherms were generated in duplicate. Isotherms with nF-CNTs, O-CNTs, and N-CNTs were generated for acetamiprid and dinotefuran. Isotherms with N-CNTs were also generated for clothianidin, thiamethoxam, and thiacloprid. All other isotherms for imidacloprid (nF/O/N-CNTs), imidacloprid urea (nF/O/N-CNTs), and desnitro-imidacloprid (nF/O/N-CNTs) were previously published. Similarly, nF-CNT and O-CNT isotherms for clothianidin, thiamethoxam, and thiacloprid were previously published. All previously published isotherm data were re-analyzed here with the Freundlich sorption model with sorption parameters provided in **Table S.2** with standard errors.

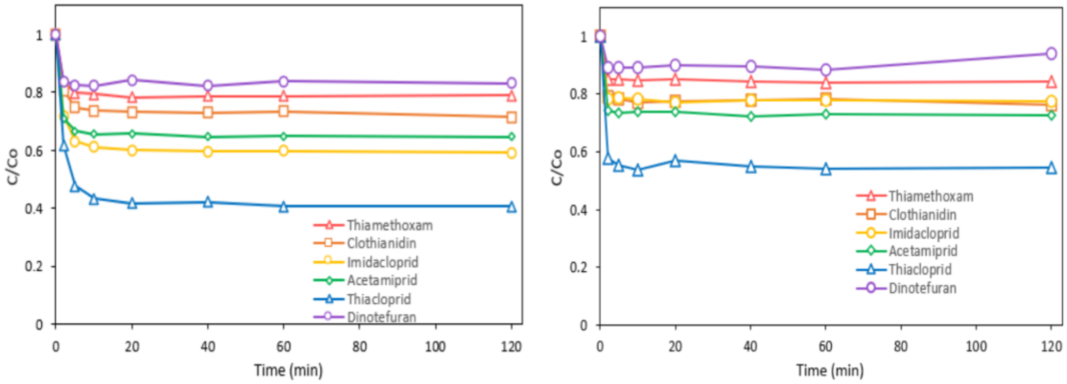

Figure S.3: Sorption kinetics of neonicotinoids to the non-functionalized carbon nanotubes (CNTs) [left panel] and COOH functionalized CNTs [right panel], demonstrating that equilibrium is rapidly achieved.

Table S.3: Neonicotinoid proton dissociation at environmentally relevant pH. Literature pKa values indicate the neonicotinoids included in this study are likely neutral at environmentally relevant pH values (pH 5-11). Dissociation states of imidacloprid are provided at the end of the table as an example reference (figure from Tomzawa et al. 2003).[3] Question marks (?) indicates instances where there is no available data in the literature, of what the authors could find, regarding proton dissociation indicate instances where it is yet unknown whether dissociation occurs at a higher/lower pH. N/A is entered where a given pKa is not available.

**Compound**	**pKa1**	**pKa2**	**Source**
Imidacloprid	1.56	11.12	https://doi.org/10.1021/bi0300130 https://onlinelibrary.wiley.com/doi/epdf/10.1002/%28SICI%291096-9063%28199607%2947%3A3%3C265%3A%3AAID-PS416%3E3.0.CO%3B2-F
imidacloprid urea	2.3	11.6	http://pubs.acs.org/doi/abs/10.1021/jf061670c
clothianidin	N/A	11.09	https://www3.epa.gov/pesticides/chem_search/cleared_reviews/csr_PC-044309_5-Mar-09_a.pdf
thiacloprid	No Dissociation		https://link.springer.com/content/pdf/10.1007/s11356-014-3332-7.pdf
dinotefuran	N/A	12.6	https://www.fs.fed.us/foresthealth/pesticide/pdfs/0521803b_Dinotefuran.pdf
desnitro-imidacloprid	No Reported Values		No Reported Values
thiamethoxam	No Dissociation		https://link.springer.com/content/pdf/10.1007/s11356-014-3332-7.pdf
acetamiprid	0.7	N/A	https://link.springer.com/content/pdf/10.1007/s11356-014-3332-7.pdf https://www3.epa.gov/pesticides/chem_search/reg_actions/registration/fs_PC-099050_15-Mar-02.pdf
thiacloprid amide	No Reported Values		No Reported Values

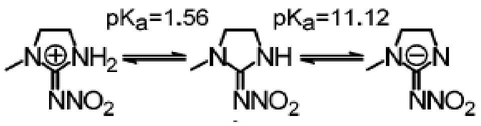

Table S.4: QSAR descriptors as obtained from Spartan'18 parallel suite computations. Descriptors A-AF were obtained directly from Spartan. Descriptors AG-AR were calculated based on each compounds chemical formula. Each descriptor is accompanied by units (if applicable), a short description of what each parameter means, and the tab each descriptor can be found in within the Spartan program.

Decriptor label	QSAR Descriptor	Units	Description	Tab
A	Energy (au)	au (arbitrary units)	A molecule's total energy (Hartree-Fock molecular orbital, density functional, Moller Plesset, and Post Hartree-Fock calculations).	molecule
B	E HOMO (eV)	eV	Energy of the highest occupied molecular orbital	molecule
C	Dipole (debye)	debye	Measure of charge separation.	molecule
D	Tautomers		Number of isomers resulting from proton transfer among nitrogen, phosphorous, oxygen, and sulfur atoms.	molecule
E	E LUMO (eV)	eV	Energy of the lowest unoccupied molecular orbital.	molecule
F	Molecular Wt. (amu)	amu (atomic mass units)	Molecular weight a molecule	molecule
G	Conformers		Number of singly-bonded conformers in a systematic equilibrium conformer or conformer distribution calculation.	molecule
H	CPK Area (Å²)	Å²	Surface area of a molecule	QSAR-CPK model
I	CPK Volume (Å³)	Å³	Volume of a molecule	QSAR-CPK model
J	PSA (Å²)	Å²	Polar surface area of a molecule determined by the summation of the area of nitrogen, oxygen, and hydrogen atoms attached to nitrogen and oxygen.	QSAR-CPK model
K	CPK Ovality		Measurment of the deviation from a spherical shape	QSAR-CPK model
L	Acc. Area (Å²)	Å²	Accessible surface area based on the electron density surface, determined with a 1.0 angstrom radius probe	QSAR-computed electron density
M	Min ElPot (kJ/mol)	kJ/mol	Minimum electrostatic potential at the electron density surface	QSAR-computed electron density
N	Max ElPot (kJ/mol)	kJ/mol	Maximum electrostatic potential at the electron density surface.	QSAR-computed electron density
O	Polar Area(75) (Å²)	Å²	Area of electron density for which the absolute value of the electrostatic potential is above and below 75 kJ/mol in the electrostatic potential map	QSAR-computed electron density
P	Acc. Polar Area(75) (Å²)	Å²	Accessible area of electron density for which the absolute value of the electrostatic potential is above and below 75, kJ/mol in the electrostatic potential map	QSAR-computed electron density
Q	Min LocIonPot (ev)		Minimum local ionization potential at the electron density surface	QSAR-computed electron density
R	Log P		water/octanol partition coefficient, calculated with the Ghose-Crippen method	QSAR tab
S	HBD Count		Number of hydrogen bond donors	QSAR tab
T	Polarizability		Empirically estimated polarizability	QSAR tab
U	HBA Count		Number of hydrogen bond acceptors	QSAR tab
V	Property Min(Surface1) kJ	kJ	Minimum displayed electron ionization energy mapped between -250 and 250 kJ and plot with an iso val of 99.8% (0.0006 e-/arbitrary unit volume).	surface properites
W	Property Max(Surface1) kJ	kJ	Maximum displayed electron ionization energy mapped between -250 and 250 kJ and plot with an iso val of 99.8% (0.0006 e-/arbitrary unit volume).	surface properites
X	Surf Area(Surface1)(Å²)	Å²	Surface area in square angstroms	surface properites
Y	Acc. Area(Surface1)(Å²)	Å²	Accessible area on the electron density surface determined by examining the surface with a 1.0 angstrom radius probe.	surface properites
Z	Polar Area(Surface1)(Å²)	Å²	Polar area on the electron density surface determined by examining the surface with a 1.0 angstrom radius probe	surface properites
AA	Surf Volume(Surface1)(Å³)	Å³	Surface volume in cubic angstroms	surface properites
AB	Acc. P-Area(Surface1)(Å²)	Å²	Accessible polar area on the electron density surface determined by examining the surface with a 1.0 angstrom radius probe	surface properites
AC	Net EI	kJ/mol		
AD	Diff in property max/min	kJ		
AE	HOMO-LUMO Gap	eV		
AF	Acc P-Area/Acc Area			
AG	#C			from molecular formula
AH	#H			from molecular formula
AI	#N			from molecular formula
AJ	#O			from molecular formula
AK	#Cl			from molecular formula
AL	#S			from molecular formula
AM	#Rings			from structure
AN	#bonds			from molecular formula
AO	DBE			from molecular
AP	McGowan Vol (cm^3/mol)	cm³/mol		from molecular formula
AQ	C/H			from molecular formula
AR	C/O			from molecular formula

Table S.5: Experimentally determined solute parameters for the 76 pesticides reported by Tülp et al.[4] along with the literature log Kow values reported therein.

		E	S	A	B	V	log Kow
1	2-nitroanisole	0.87	1.47	0.22	0.3	1.09	1.73
2	2,4-DNT	1.17	1.27	0.07	0.51	1.206	2
3	2,4,6-TNT	1.57	1.71	0.11	0.62	1.38	2.06
4	3-nitroanisole	0.87	1.2	0.14	0.25	1.09	2.16
5	4-nitroanisole	0.87	1.32	0.2	0.24	1.09	2.03
6	Alachlor	1.17	1.19	0.05	1.17	2.14	2.77
7	amitrole	1.06	0.97	1.03	0.46	0.595	-0.16
8	Atrazine	1.51	1.23	-0.05	1.05	1.62	2.59
9	Azoxystrobin	2.24	2.08	0.15	1.88	2.917	2.5
10	bensulide	2.09	1.72	0.51	1.55	2.873	4.1
11	bromacil	1.03	1.01	0.88	0.88	1.631	1.87
12	carbamazepine	2.15	1.79	0.42	1.11	1.811	2.63
13	carbaryl	1	1.3	0.47	0.7	1.541	2.34
14	carbendiazim	2	1.69	0.56	0.7	1.361	1.51
15	chlorobenzilate	1.72	0.36	0.05	1.45	2.266	4.58
16	chlorothalonil	1.64	2.1	-0.29	0.48	1.515	2.97
17	chlorpyrifos	1.44	0.34	-0.38	1.13	2.15	5.07
18	clofentazine	2.08	0.93	0.09	1.06	2.012	3.1
19	clothianidin	2.21	2.11	0.83	1	1.576	0.6
20	cyanazine	1.73	1.67	0.21	1.19	1.774	2.15
21	cymoxanil	0.95	1.83	0.51	0.86	1.455	0.67
22	cypermethrin	1.72	0.24	0.02	1.55	2.973	5.66
23	cyproconazole	1.7	1.07	0.35	1.36	2.162	2.91
24	cyromazine	2.5	0.1	1.23	1.41	1.207	-0.16
25	desethylatrazine	1.5	1.05	0.36	1.04	1.338	1.7
26	diazepam	2.49	1.46	0.1	1.36	2.074	3.04
27	diazinon	1.04	1.01	0	1.19	2.306	3.35
28	dichlofluanid	2.43	1.21	-0.1	1.33	2.046	3.7
29	diflufenican	1.39	1	0.36	1.11	2.435	4.9
30	dimethenamid	1.27	1.28	-0.02	1.26	2.065	2.15
31	dinoseb	0.9	1.75	0.17	0.46	1.687	3.47
32	diuron	1.5	1.59	0.5	0.73	1.599	2.67
33	endosulfan	1.26	0.88	0.47	0.98	2.082	3.67
34	endrin	0.54	1.28	-0.01	0.38	2.007	4.88
35	ethofumesat	1.2	1.71	0.09	1.07	2.051	2.69
36	famoxadone	2.63	2.21	0.26	1.21	2.735	4.73
37	fenthion	1.36	1.75	-0.03	0.65	1.988	4.13
38	fluazinam	1.13	1.7	0.24	1.23	2.364	3.56
39	flucythrinate	1.69	-0.09	0.71	2.57	3.3	5.92
40	flumioxazin	0.42	2.17	0.12	1.1	2.416	2.55
41	fluorouracil	0.7	0.64	1.09	0.7	0.769	-0.89
42	flusilazole	1.47	1.76	0.33	1	2.274	3.75
43	fosthiazate	1.14	1.39	0.31	1.26	2.091	1.75
44	imiprothrin	1.65	1.37	0.15	1.53	2.463	2.9
45	ingarol/irgarol	1.52	1.52	-0.26	0.99	1.975	3.95
46	isoproturon	1.2	1.52	0.39	0.96	1.777	2.38
47	metamitron	1.89	1.49	0.37	1.15	1.5	0.69
48	metazachlor	1.62	1.48	0.11	1.37	2.087	2.13
49	methidathion	2.07	1.6	0.04	1.12	1.889	2.39
50	metolachlor	1.15	1.01	0.07	1.38	2.281	3.21
51	metoxuron	1.33	1.78	0.49	0.89	1.676	1.64
52	metribuzin	1.61	1.17	0.32	1.03	1.62	1.65
53	monouron	1.36	1.51	0.47	0.82	1.477	1.82
54	napropamide	1.51	1.44	0.03	1.22	2.246	3.36
55	nitrofen	1.78	0.82	-0.28	0.85	1.802	4.64
56	octhilinone	0.89	1.69	0.13	0.62	1.786	2.45
57	orbencarb	1.33	1.17	-0.08	0.86	1.963	3.5
58	parathion	1.33	1.76	0.05	0.72	1.998	3.83
59	phenmedipham	2.15	1.64	0.58	1.38	2.236	3.59
60	procymidone	1.45	0.51	-0.01	1.25	1.862	3.07
61	propachlor	1.12	1.41	-0.08	0.96	1.659	2.19
62	propaquizafop	1.8	1.42	-1.05	2.52	3.168	4.69
63	propioconazole	1.24	1.41	0.12	1.18	2.343	3.61
64	propoxur	0.72	1.2	0.38	0.91	1.654	1.55
65	pymetrozine	1.93	2.58	0.46	0.92	1.6	-0.18
66	sulfentrazone	2.32	1.32	0.69	1.65	2.245	0.99
67	tebutam	0.8	1.11	0.02	1.12	2.1	3.5
68	terbutryne	1.21	1.33	-0.12	0.95	1.943	3.61
69	thiazopyr	0.8	1.07	0.06	1.32	2.499	3.89
70	triclabendazole	2.84	1.67	0.39	1.03	2.243	N/A
71	triclosan	1.85	1.69	0.74	0.29	1.809	4.92
72	triflumuron	1.62	1.14	0.64	0.94	2.212	4.91
73	trifluralin	1.06	0.4	-0.1	1.35	2.204	4.73
74	trilate	1.08	0.92	0.2	0.75	2.121	4.66
75	vinclozolin	1.95	1.27	-0.35	1.36	1.845	2.74
76	zearalenone	1.75	1.57	0.84	1.25	2.463	4.12

SMILES Structures. Canonical SMILES structures for neonicotinoids and metabolites were obtained from PubChem (**Table S.6**) and used in the UFZ-LSER database to calculate the Abraham solute parameters for each neonicotinoid and subsequently calculate pp-LFER log K_{OW} and log K_F values.

Table S.6: SMILES-predicted Abraham solute parameters for neonicotinoids and neonicotinoid metabolites. Color coding provides information regarding the application of the domain in which green is indicative of a relatively good prediction, yellow indicates low similarity to chemicals within the training set/near the limits of the training set, and orange is indicative of the chemical being out of the domain of the training set and a structural outlier.

	Query	E_{QSPR}	S_{QSPR}	A_{QSPR}	B_{QSPR}	V	$warning_E$
imidacloprid	C1CN(C(=N[N+](=O)[O-])N1)CC2=CN=C(C=C2)Cl	1.73	1.9	0.17	1.49	1.6833	
clothianidin	CNC(=N[N+](=O)[O-])NCC1=CN=C(S1)Cl	1.6	1.45	0.37	1.58	1.5757	low similarity
thiamethoxam	CN1COCN(C1=N[N+](=O)[O-])CC2=CN=C(S2)Cl	1.86	1.89	0	1.73	1.8076	
thiacloprid	C1CSC(=NC#N)N1CC2=CN=C(C=C2)Cl	1.91	2.11	0.05	1.37	1.7275	
acetamiprid	CC(=NC#N)N(C)CC1=CN=C(C=C1)Cl	1.4	1.57	0.05	1.21	1.6726	
dinotefuran	C1COCC1CN=C(N)N[N+](=O)[O-]	0.92	1.39	0.37	1.41	1.3347	high leverage low similarity
desnitro-imidacloprid	C1CN(C(=N1)N)CC2=CN=C(C=C2)C	1.43	1.54	0.27	1.63	1.5276	high leverage
imidacloprid urea	C1CN(C(=O)N1)CC2=CN=C(C=C2)Cl	1.34	2.05	0.34	1.09	1.468	
thiacloprid amide	C1CSC(=NC(=O)N)N1CC2=CN=C(C=C2)Cl	2.12	2.17	0.39	1.72	1.8292	out of domain

McGowan Volume calculations**:** The McGowan volume was calculated as follows in **Equation S.2**:

Equation S.2

$$V_x = \left(\frac{\sum all\ atom\ contributions\ - \sum 6.56 * \#\ Bonds}{100}\right)$$

Where all V_x is the molar volume (mL/mol), total atom contributions are the number of each atom multiplied by the atomic volume of each atom, and number of bonds is calculated following **Equation S.3**.[5–7] Volumes are typically adjusted by a factor of 100 for use in pp-LFERs/LSERs and thus, was adjusted here as well.

Equation S.3

$$\#Bonds = N - 1 + \#Rings$$

Where N is the number of atoms.

Model Development/p-Value Exception. All parameters in finalized models had a p-value below 0.1 with the exception of the dipole parameter in the neonicotinoid CNT multiple linear regression (p=0.1513). In this instance, the p-value was deemed acceptable considering three neonicotinoids were omitted from parameter fitting (thiamethoxam, acetamiprid, and thiacloprid amide). When thiamethoxam was included in the model development (model fitting) the dipole parameter dropped to a p-value of p=0.0525 (**Table S.7**) with little impact on the parameter coefficients. Thus, we deemed the dipole parameter (p=0.1513) for our CNT multiple linear regression acceptable for the purposes of modeling neonicotinoid sorption.

Table S.7: Multiple linear regression model parameters, coefficients, and internal validation information for the CNT-QSAR model chosen for analysis including thiamethoxam in model fitting rather than as a means to externally validate the model.

Descriptor	Coefficient	Standard Error	t-test	p-value	VIF
Intercept	-2.203	0.6373	3.457	0.0086	
Property Max(Surface2) kJ	0.003011	0.0009838	3.060	0.0156	1.837
Molecular Wt. (amu)	0.01502	0.003274	4.588	0.0018	4.905
HBA Count	-0.1976	0.06415	3.080	0.0151	4.725
Dipole (debye)	-0.09779	0.04298	2.275	0.0525	6.776
Acc. P-Area(Surface2)(Å²)	0.01284	0.004556	2.818	0.0225	6.461
$R^2 = 0.8635$, $R_{adj}^2 = 0.7782$, *RMSE=0.1298, n=14*					

Analysis. All samples were analyzed on an Agilent 1260 Infinity liquid chromatograph with an Agilent 6460 triple quadrupole mass spectrometer (LC-MS/MS) with electrospray ionization in positive ionization multiple reaction monitoring mode (MRM) (**Table S.8**, **Table S.9**) identical to our previous methods.[8,9] Analytes were separated on an Agilent Zorbax eclipse plus C18 column (4.6 mm x 150 mm x 5 µm) with a Zorbax eclipse plus C18 guard column (4.6 mm x 12.5 mm x 5 µm). An injection volume of 20 µL was loaded onto the column preheated to 50 °C. The mobile phases contained 0.1% formic acid in (A) water (77.5%) and (B) acetonitrile (22.5%) with a flow rate of 0.8 mL min^{-1}. Lower limits of detection (LLD) were previously reported[9] and are provided in **Table S.9** with MRM transitions (a quantitative ion for sample quantification and qualitative ion for compound verification). Peak analysis was done with Agilent MassHunter Qualitative Analysis software (version B.06.00) and a five-point isotope-normalized (deuterated imidacloprid) external calibration curve accounted for surrogate recovery and differential ionization during quantification and was linear throughout range.

Table S.8: Mass spectrometer settings. Agilent 6460 Triple quadrupole mass spectrometer instrumental settings are provided below. These settings were used for all LC-MS/MS analysis.

**MS/MS Parameters**	
Gas Temperature (N2)	300 °C
Gas Flow	5 L/min
Nebulizer Pressure	45 psi
Sheath Gas Temperature	250 °C
Sheath Gas Flow	11 L/min
Capillary Voltage (+)/(-)	3500/3500 V
Nozzle Voltage (+)/(-)	500/500 V

Table S.9: MRM transitions. Agilent 6460 Triple quadrupole mass spectrometer neonicotinoid and neonicotinoid metabolite mass transitions used for analysis. The quantitation ion (quant ion) was used for quantifying neonicotinoid and metabolite concentrations. The qualitative ion (qual ion) was used to verify compound identity. The collision energies for each MRM is provided in parentheses following the quantitative and qualitative ions. The LLD was determined by methods previously reported and outlined by the Standards Method 1030 E Method Detection Level.[10] A standard containing imidacloprid (30 nM), clothianidin (20 nM), thiamethoxam (0.5 nM), thiacloprid (15 nM), and imidacloprid urea and desnitro- imidacloprid (1 nM each) were injected 7 consecutive times on the LC-MS/MS. The standard deviations of the measured concentrations were calculated for each compound and LLD calculated by 2*SD*1.654. Multiplying the standard deviation by two-times the t-statistic reduces the probability of a type I error.

Compound	RT (min)	Parent Ion (m/z)	Quant Ion (m/z) (Collision Energy, V)	Qual Ion (m/z) (Collision Energy, V)	Fragment Voltage (V)	Dwell Time (ms)	Polarity	Accelerator Voltage (V)	LLD (ng/L)
Desnitro-Imidacloprid	2.4	211.1	126 (22)	90.03 (36)	63	200	Positive	4	25.6
Dinotefuran	3.2	203.1	129 (10)	114.1 (10)	80	200	Positive	4	383
Thiamethoxam	4.8	392.03	211 (8)	181 (20)	63	200	Positive	4	81.2
d_3-Thiamethoxam	4.9	295.05	214 (8)	131.9 (20)	71	200	Positive	4	N/A
Imidacloprid Urea	5.1	212.06	128 (16)	99.1 (16)	76	200	Positive	4	56.8
Clothianidin	6.8	250.02	169.1 (8)	131.9 (12)	67	200	Positive	4	488
Thiacloprid amide	7.2	271.04	126 (25)	228 (13)	90	200	Positive	4	92.8
Imidacloprid	7.9	256.06	213 (8)	175.1 (12)	67	200	Positive	4	428
d_4-Imidacloprid	7.8	260.09	213 (12)	179.1 (16)	59	200	Positive	4	N/A
Acetamiprid	11.6	223	125.9 (20)	56.1 (15)	120	200	Positive	4	166
Thiacloprid	18	253	126 (20)	90 (40)	106	200	Positive	4	2101

QA/QC. Amber or foil-covered glass serum vials were used in bench experiments to minimize photolysis or evaporative losses. All isotherms where sodium azide was added to control for microbial growth were conducted alongside no-black carbon controls containing sodium azide (where no loss due to azide was observed for any of the neonicotinoids). All samples were spiked with d_4-imidacloprid to account for instrumental variability upon analysis. Samples not immediately analyzed were stored at -20 °C until analysis was performed. Samples were diluted ten-fold using Optima grade LCMS water prior to analysis on any instrument to work within the linear calibration range.

SUPPLEMENTAL RESULTS and DISCUSSION

Table S.10: QSAR data obtained in Spartan '18 for the 76 pesticides outlined in **Table S.5** from Tülp et al.[4]

		A	B	C	D	E	F	G	H	I	J	K
1	2-nitroanisole	-551.096	-8.53	7.47	0	-0.4	153.137	2	169.5	147.53	44.557	1.26
2	2,4-DNT	-680.354	-9.73	6.38	0	-0.9	182.135	1	183.51	159.67	76.13	1.29
3	2,4,6-TNT	-884.779	-10.2	1.77	0	-1.23	227.132	1	207.25	180.87	113.747	1.34
4	3-nitroanisole	-551.103	-8.47	7.79	0	-0.62	153.137	2	169.86	147.45	45.591	1.26
5	4-nitroanisole	-551.107	-8.57	7.64	0	-0.49	153.137	1	169.89	147.43	45.565	1.26
6	Alachlor	-1210.71	-8.59	5.74	0	1.2	269.772	432	305.4	283.53	20.708	1.46
7	amitrole	-297.533	-8.15	3.07	4	2.5	84.082	1	100.75	76.62	58.972	1.15
8	Atrazine	-1047.1	-8.59	5.93	8	1.23	215.688	144	245.62	209.7	41.894	1.44
9	Azoxystrobin	-1388.49	-8.39	9.33	0	0.26	403.394	192	414.99	397.09	66.049	1.59
10	bensulide	-2439.21	-8.73	12.15	2	0.83	397.521	39366	422	371.15	60.538	1.69
11	bromacil	-3184.54	-8.43	5.87	2	0.8	261.119	18	234.21	210.92	37.218	1.37
12	carbamazepine	-763.337	-7.91	5.56	1	0.45	236.274	18	254.33	245.29	37.187	1.34
13	carbaryl	-668.923	-7.96	7.1	1	0.53	201.225	12	227.36	210.21	29.986	1.33
14	carbendiazim	-662.925	-7.8	4.55	4	1.4	191.19	4	208.01	184.86	48.824	1.33
15	chlorobenzilate	-1763.88	-8.58	2.32	0	0.82	325.191	81	316.56	300.24	37.46	1.46
16	chlorothalonil	-2254.88	-9.58	3.82	0	-0.97	265.914	1	210.46	190.81	30.668	1.31
17	chlorpyrifos	-2749.94	-8.94	4.07	0	0.27	350.59	486	320.57	275.14	29.6	1.57
18	clofentazine	-1677.35	-8.81	4.24	0	-1.07	303.152	4	285.52	266.89	37.575	1.42
19	clothianidin	-1515.69	-8.88	9.89	4	0.61	249.682	36	237.96	201.43	77.636	1.43
20	cyanazine	-1139.31	-8.76	9.43	8	1.05	240.698	144	261.43	228.5	55.277	1.45
21	cymoxanil	-717.179	-9.19	14	4	-0.69	198.182	576	230.89	191.49	78.435	1.44
22	cypermethrin	-2050.16	-8.15	4.91	0	0.96	416.304	108	429.11	402.58	40.933	1.63
23	cyproconazole	-1281.82	-8.63	1.79	0	1.44	291.782	243	303.47	291.64	31.726	1.43
24	cyromazine	-563.034	-8.34	1.49	16	2.24	166.188	6	192.4	160.41	82.369	1.35
25	desethylatrazine	-968.504	-8.68	5.86	8	1.24	187.634	12	203.3	170.75	56.805	1.37
26	diazepam	-1262.2	-8.31	4.79	0	0.22	284.746	18	287.53	277.98	21.778	1.4
27	diazinon	-1544.47	-9.14	8.66	0	0.97	304.351	972	342.83	300.74	33.603	1.58
28	dichlofluanid	-2425.76	-8.93	3.6	0	0.81	333.235	162	302.57	266.67	36.105	1.51
29	diflufenican	-1489.38	-8.56	7.08	1	0.62	394.299	8	363.84	342.37	37.619	1.54
30	dimethenamid	-1531.48	-8.31	7.87	0	1.45	275.8	324	293.35	270.2	21.001	1.45
31	dinoseb	-873.471	-8.99	5.39	2	-1.04	240.215	12	244.87	220.39	89.189	1.39
32	diuron	-1453.93	-8.07	3.63	1	1.24	233.098	4	231.36	208.09	25.612	1.36
33	endosulfan	-3731.15	-8.64	21.22	0	-1.24	406.928	162	295.08	269.6	35.766	1.46
34	endrin	-3298.13	-9.03	5.38	0	0.88	380.913	1	275.46	266.65	10.073	1.37
35	ethofumesat	-1280.2	-8.05	2.9	0	1.49	286.348	432	307.22	275.57	56.421	1.5
36	famoxadone	-1259.2	-8.03	3.2	0	1	374.396	12	392.78	376.28	55.82	1.56
37	fenthion	-1753.33	-8.44	4.83	0	1.19	278.333	108	297.17	259.76	22.802	1.51
38	fluazinam	-2536.44	-8.63	2	3	-1.28	465.093	4	347.57	320.91	84.246	1.53
39	flucythrinate	-1559.48	-8.2	6.38	0	0.97	451.469	648	475.15	451.29	48.49	1.67
40	flumioxazin	-1242.89	-8.06	5.09	0	-0.49	354.337	48	350.34	335.59	52.066	1.5
41	fluorouracil	-513.901	-8.59	5.54	5	0.83	130.078	2	128.25	105.44	51.727	1.19
42	flusilazole	-1271.87	-8.63	3.73	0	1.3	315.399	9	334.75	314.86	19.479	1.5
43	fosthiazate	-1847.01	-8.75	7.38	0	1.01	299.352	2187	313.44	271.44	48.727	1.55
44	imiprothrin	-1071.1	-8.32	4.84	0	1.54	318.373	243	373.95	339.48	50.686	1.59
45	ingarol/irgarol	-1102.36	-8.37	4.22	8	1.46	253.374	96	297.04	262.67	39.238	1.5
46	isoproturon	-652.683	-7.75	4.65	1	1.8	206.289	4	258.91	235.6	25.76	1.4
47	metamitron	-681.063	-8.43	4.07	0	-0.12	202.217	3	221.23	200.07	59.413	1.34
48	metazachlor	-1242.56	-8.59	5.97	0	1.2	277.755	54	292.86	279.22	24.065	1.42
49	methidathion	-2181.35	-8.46	6.06	0	1.29	302.336	729	287.87	243.94	48.355	1.52
50	metolachlor	-1250	-8.53	5.36	0	1.25	283.799	648	316.04	300.77	20.835	1.46
51	metoxuron	-1108.83	-7.95	5.59	1	1.41	228.679	8	247.73	222.03	32.965	1.4
52	metribuzin	-1004.94	-9.99	13.75	1	-5.12	214.293	2	235.29	207.55	56.055	1.39
53	monouron	-994.35	-7.93	3.01	1	1.51	198.653	2	216.68	194.64	25.672	1.33
54	napropamide	-865.424	-7.79	6.92	0	0.68	271.36	324	319.5	302.77	21.461	1.47
55	nitrofen	-1661.94	-8.87	5.67	0	-0.58	284.098	2	262.79	240.14	45.157	1.41
56	octhilinone	-958.603	-8.02	4.97	0	1.09	213.345	2187	270.13	235.68	16.096	1.46
57	orbencarb	-1455.09	-8.63	3.9	0	1.27	257.785	324	285.89	258.06	13.671	1.46
58	parathion	-1559.61	-9.1	10.23	0	-0.7	291.264	243	302.18	262.08	60.179	1.53
59	phenmedipham	-1029.48	-8.03	4.96	3	1.55	300.314	32	331.7	303.28	59.692	1.52
60	procymidone	-1627.38	-9.05	5.6	0	0.87	284.142	1	275.06	253.6	28.945	1.42
61	propachlor	-1017.62	-8.94	7.7	0	1.47	211.692	18	239.24	219.28	14.48	1.36
62	propaquizafop	-1851.67	-8.14	6.98	0	-0.34	443.887	2916	463.25	427.73	63.214	1.69
63	propioconazole	-1816.63	-8.92	3.78	0	1.01	342.226	324	340.81	314.51	31.694	1.52
64	propoxur	-708.438	-8.05	3.29	1	1.79	209.245	24	250.74	222.87	37.231	1.41
65	pymetrozine	-736.394	-8.14	1.56	1	0.26	217.232	24	239.01	214.01	52.885	1.38
66	sulfentrazone	-2387.74	-8.56	7.93	2	0.67	387.194	72	334.6	297.83	71.779	1.55
67	tebutam	-715.242	-8.43	4.66	0	1.78	233.355	18	285.95	276.42	12.995	1.39
68	terbutryne	-1064.31	-8.42	4.29	8	1.53	241.363	96	286.64	251.31	39.1	1.49
69	thiazopyr	-1776.9	-8.57	8.84	0	0	396.38	48	364.36	340.25	33.645	1.55
70	triclabendazole	-2502.13	-7.89	8.54	1	0.9	359.664	8	318.47	295.5	25.893	1.48
71	triclosan	-1992.28	-8.3	1.51	0	1.06	289.545	8	260.54	239.3	24.245	1.4
72	triflumuron	-1672.19	-8.26	10.82	4	0.53	358.703	32	331.67	303.6	53.503	1.52
73	trifluralin	-1269.13	-8.23	0.85	0	-0.77	335.282	81	327.98	296.74	75.809	1.52
74	trilate	-2299.24	-8.71	5.14	0	1.08	304.669	486	301.65	269.54	11.822	1.49
75	vinclozolin	-1663.27	-9.15	3.88	0	0.71	286.114	3	271.16	247.57	36.029	1.42
76	zearalenone	-1074.98	-7.98	7.98	2	0.47	318.369	472392	331.68	324.76	66.694	1.45

		L	M	N	O	P	Q	R	S	T	U	V	W	X	Y	Z
1	2-nitroanisole	130.8	-220.22	182.66	92.03	71.97	11.73	-0.12	0	51.42	4	-193.78	138.24	190.65	166.73	58.85
2	2,4-DNT	139	-164.69	202.34	105.45	89.51	12.42	0.34	0	52.24	6	-140.49	158.27	201.43	169.18	47.76
3	2,4,6-TNT	149.9	-128.37	199.41	117.53	89.28	13.45	-0.35	0	53.93	9	-108.12	152.83	222.42	176.29	42.39
4	3-nitroanisole	132.24	-199.2	185.62	83.4	68.87	11.86	-0.12	0	51.48	4	-174.77	140.64	191.57	167.44	49.23
5	4-nitroanisole	131.87	-211.67	184.49	82.27	68.08	11.77	-0.12	0	51.42	4	-186.95	139.91	191.49	167.61	53.62
6	Alachlor	186.06	-246.22	113.06	77.17	48.51	11.53	1.83	0	62.07	3	-206.76	78.65	320.04	242.56	30.81
7	amitrole	90.68	-223.19	319.63	68.72	62.67	10.56	-0.42	0	45.08	2	-169.56	233.22	123.23	116.04	50.7
8	Atrazine	173.02	-216.05	240.12	88.63	60.29	11.14	2.82	0	56.07	3	-171.05	164.68	282.68	233.86	48.12
9	Azoxystrobin	253.91	-294.06	165.83	159.91	99.73	11.14	0.91	0	71.55	7	-256.76	125.02	410.46	305.78	90.15
10	bensulide	247.85	-239.66	267.59	203.33	131.07	10.01		1	69.23	8	-207.9	196.78	423.9	316.08	106.11
11	bromacil	158.46	-255.55	348.45	86.79	61.18	11.21	0.37	1	56.31	4	-216.52	271.11	255.61	201.26	62.01
12	carbamazepine	181.37	-305.05	212.05	93.08	71.09	10.98	-0.32	1	59.3	3	-259.14	144.93	275.06	225.06	40.71
13	carbaryl	168.18	-276.96	247.54	82.36	61.51	11.46	0.4	1	56.42	2	-232.7	175.48	251.97	212.94	38.06
14	carbendiazim	164.2	-226.21	254.97	94.91	82.18	11.02	-0.34	0	54.2	2	-175.13	173.79	234.57	206.29	35.34
15	chlorobenzilate	215.02	-156.25	173.17	86.23	57.23	11.99	0.97	1	63.51	3	-130.06	109.48	342.91	269.06	10.87
16	chlorothalonil	168.53	-175.86	160.31	91.75	82.17	13.02	0.45	0	54.82	2	-144.72	122.02	241.97	201.65	28.47
17	chlorpyrifos	214.46	-200.43	189.71	89.04	54.26	10.3		0	61.52	5	-169.58	132.11	354.98	269.97	42.15
18	clofentazine	220	-215.75	141.54	109.37	83.59	11.95	3.36	0	61.2	4	-181.48	99.93	316.65	269.15	33.26
19	clothianidin	171.84	-279.84	416.26	144.3	102.27	11.48	1.54	0	55.48	6	-248.01	351.67	268.54	213.42	96.33
20	cyanazine	182.55	-255.66	290.03	138.49	98.66	11.31	2.79	0	57.6	4	-215.13	210.77	298.09	235.73	83.83
21	cymoxanil	168.23	-364.03	394.7	155.25	115.19	11.96	0.28	2	54.9	7	-316.21	320.37	263.58	218.98	131.44
22	cypermethrin	287.09	-222.7	157.84	113.04	84.49	11.51	3.14	0	71.88	3	-182.65	112.02	469.01	354.62	37.86
23	cyproconazole	202.55	-218.2	189.33	86.66	62.28	11.2	1.61	1	62.66	3	-170.14	121.75	320.76	253.1	17.21
24	cyromazine	143.01	-217.56	201.82	89.96	68.23	10.83	0.39	0	51.9	3	-155.26	134.21	217.34	183.76	49.5
25	desethylatrazine	149.27	-207.75	266.66	86.51	64.99	11.17	1.96	0	52.89	3	-165.94	189.15	234.47	194.93	54.06
26	diazepam	207	-236.16	178.63	89.89	66.14	10.84	0.74	0	61.91	3	-197.79	132.91	315.31	257.11	43.34
27	diazinon	208.96	-247.28	164.73	145.55	94.69	10.21		0	63.39	6	-211.2	126.03	366.27	277.84	71.1
28	dichlofluanid	179.26	-208.47	132.2	91.33	61.12	11.13		0	60.71	6	-181.15	101.44	298.28	218.15	37.86
29	diflufenican	235	-223.01	280.45	123.5	90.39	11.36	0.25	1	66.98	4	-192.34	218.91	373.94	273.94	53.19
30	dimethenamid	183.07	-283.82	162.42	117.7	76.36	10.96	0.32	0	60.99	4	-240.75	115.38	313.29	235.38	36.97
31	dinoseb	170	-172.22	228.78	86.8	66.86	12.44	0.42	1	57.37	7	-147.62	157.89	264.82	210.17	25.87
32	diuron	165.76	-225.45	250.95	86.35	61.55	11.8	-0.37	1	56.06	3	-187.45	182.54	258.29	207.18	29.51
33	endosulfan	176.76	-415.01	361.17	233.53	145.17	9.76		0	61.5	4	-364.98	318.9	303.3	211.72	208.9
34	endrin	171.38	-130.9	149.49	72.3	47.55	12.61	2.38	0	60.67	1	-106.44	115.37	287.02	211.11	9.84
35	ethofumesat	191.99	-216.37	164.45	101.75	71.3	11.59	0.37	0	61.48	6	-183.98	115.74	330.98	248.07	52.58
36	famoxadone	262.12	-223.71	257.87	127.55	97.03	11.37	0.67	1	69.77	5	-192.52	186.74	413.55	316.8	50.65
37	fenthion	197.15	-206	137.92	122.27	93.72	9.72		0	60.18	5	-166.68	95.96	319.05	253.16	46.74
38	fluazinam	214.21	-135.45	178.71	101.69	62.42	11.76	0.93	0	65.67	7	-119.16	143.61	350.84	240.01	28.3
39	flucythrinate	305.85	-219.23	169.59	137.83	101.91	11.58	2.56	0	75.82	4	-181.22	119.38	502.23	376.69	40.76
40	flumioxazin	230.39	-199.44	190.53	123.93	97.26	11.29	-2.28	0	66.81	6	-168.84	134.04	371.3	284.36	49.62
41	fluorouracil	102.65	-228.11	362.59	80.68	67.87	11.89	-1.31	2	47.71	4	-195.8	292.39	141.97	123.72	63.08
42	flusilazole	219.64	-230.72	202.85	115.26	80.71	11.14		0	64.58	2	-183.93	165.41	342.1	263.15	45.98
43	fosthiazate	184.48	-360.12	177.2	127.8	76.63	10.13		0	61.09	7	-313.1	132.84	328.51	247	67.54
44	imiprothrin	234.17	-264.75	203.22	112.59	75.53	10.47	1.69	0	66.59	5	-235.31	138.99	394.6	288.9	61.5
45	ingarol/irgarol	196.52	-261.84	207.08	79.15	54.44	10.08	3.25	0	60.37	4	-206.19	137.17	321.51	257.32	36.91
46	isoproturon	171.69	-257.32	188.48	51.68	39.7	11.31	0.83	1	58.24	3	-212.42	117.77	285.94	224.14	23.74
47	metamitron	170.08	-236.26	257.46	95.71	77.29	11.1	0.99	1	55.64	4	-199.41	185.55	244.51	213.02	53.64
48	metazachlor	190.56	-250.6	188.96	91.77	64.89	11.13	1.31	0	61.72	4	-212.5	144.15	304.88	237.31	36.69
49	methidathion	178.74	-264.72	175.82	148.61	108.02	10.16		0	58.86	9	-236.07	135.04	294.38	225.02	94.41
50	metolachlor	195.15	-269.43	152.37	86.73	56.25	11.44	1.45	0	63.47	3	-229.08	102.88	329.26	252.77	32.09
51	metoxuron	170.31	-239.54	215.87	97.13	72.03	11.66	-1.21	1	57.18	4	-199.49	145.6	271.93	214.01	31.84
52	metribuzin	156.89	501.85	1085.27	222.36	156.89	12	2.98	0	57.06	6	469.67	951.58	255.23	200.38	255.23
53	monouron	156.3	-236.07	226.33	74.41	56.09	11.64	-0.23	1	54.94	3	-195.12	154.32	243.88	199.32	24.66
54	napropamide	198.47	-293.43	135.32	104.73	64.13	11.32	0.88	0	63.94	3	-253.36	97.59	330.69	253.42	36.71
55	nitrofen	192.67	-194.9	182.08	84.58	64.36	12.03	0.09	0	58.9	4	-172.01	135.82	289.55	234.66	42.94
56	octhilinone	178.5	-267.08	202.25	49.24	35.68	10.69		0	58.34	3	-218.32	139.01	310.97	247.18	33.49
57	orbencarb	193.29	-221.1	120.37	88.11	59.63	10.22	2.14	0	59.98	3	-183.34	88.61	310.96	254.97	22.36
58	parathion	200.17	-191.73	164.16	163.93	122.02	10.2		0	60.65	4	-166.74	131.44	327.94	256.94	85.22
59	phenmedipham	238.64	-238.18	279.97	109.87	77.24	11.26	-0.2	2	63.72	4	-200.69	203.33	362.59	299.72	54.71
60	procymidone	188.19	-183.64	171.64	91.54	61.55	12.26	1.04	0	59.61	3	-152.42	135.38	304.68	231.94	38.14
61	propachlor	161.25	-293.56	134.62	95.73	69.3	11.81	1.18	0	56.71	2	-246.05	92.96	263.51	211.55	32.84
62	propaquizafop	320.13	-234.67	177.92	124.17	87.42	11.29	-0.35	0	74.23	7	-195.12	134.17	510.7	401.48	45.19
63	propioconazole	220.57	-232.99	139.53	88.34	60.37	11.18	2.05	0	64.55	4	-185.29	97.31	361.29	275.39	26.23
64	propoxur	167.37	-275.37	239.65	85.28	53.51	11.42	0.03	0	57.13	3	-231.19	166.29	275.84	217.56	36.32
65	pymetrozine	183.26	-224.43	210.84	93.98	75.94	10.71	-0.28	0	56.75	6	-173.27	137.73	267.33	233.59	32.03
66	sulfentrazone	217.19	-230.45	250.33	149.21	107.82	12.09	-1.25	1	63.36	7	-201.14	185.24	348.4	257.22	85.45
67	tebutam	178.02	-257.96	119.49	50.72	29.31	11.53	3.03	0	61.39	2	-212.94	84.2	303	235.97	19.33
68	terbutryne	194.21	-241.88	210.14	78.96	50.12	10.07	3.29	0	59.42	4	-189.03	138.59	321.98	260.26	36.57
69	thiazopyr	204.51	-237.1	147.41	157.45	108.94	10.21	3.46	0	66.95	4	-205	114	365.45	245.2	49.53
70	triclabendazole	233.1	-176.47	371.57	126.71	85.66	10.28	-0.3	0	63.27	3	-152.79	287.67	352.6	285.49	48.21
71	triclosan	195.31	-112.86	290.8	52.69	35.44	11.79	-0.44	1	58.58	2	-85.93	203.29	294.44	239.95	10.19
72	triflumuron	227.76	-345.1	421.12	118	80.15	11.72		2	63.93	5	-304.08	355.44	349.25	271.88	78.58
73	trifluralin	186.08	-133.84	178.13	73.81	45.87	12.65	1.6	0	63.68	7	-112.38	129.39	331.42	225.63	15.54
74	trilate	190.78	-215.21	127.31	67.05	33.61	10.34	3.21	0	60.93	3	-176.77	95.56	323.62	243.44	11.87
75	vinclozolin	186.87	-191.5	160.32	91.23	63.21	11.21	0.83	0	59.13	3	-160.84	118.07	300.05	230.53	25.24
76	zearalenone	209.39	-199.98	352.93	147.25	104.72	10.84	0.82	2	65.72	4	-168.39	269.18	347.15	265.49	61.44

		AA	AB	AC	AD	AE	AF	AG	AH	AI	AJ	AK	AL	AM	AN	AO	AP	AQ	AR
1	2-nitroanisole	198.09	48.19	402.88	332.02	-8.13	0.28903	7	7	1	3	0	0	1	18	5	1.0867	1	2.33
2	2,4-DNT	210.12	40.5	367.03	298.76	-8.83	0.23939	7	6	2	4	0	0	1	19	6	1.2022	1.167	1.75
3	2,4,6-TNT	232.6	35.96	327.78	260.95	-8.97	0.203982	7	5	3	6	0	0	1	21	7	1.3764	1.4	1.17
4	3-nitroanisole	198.48	42.96	384.82	315.41	-7.85	0.25657	7	7	1	3	0	0	1	18	5	1.0867	1	2.33
5	4-nitroanisole	198.3	48.57	396.16	326.86	-8.08	0.28978	7	7	1	3	0	0	1	18	5	1.0867	1	2.33
6	Alachlor	386.54	19.83	359.28	285.41	-9.79	0.081753	14	20	1	2	1	0	1	38	5	2.1332	0.7	7
7	amitrole	114.74	48.56	542.82	402.78	-10.65	0.418476	2	4	4	0	0	0	1	10	3	0.594	0.5	
8	Atrazine	307.43	31.62	456.17	335.73	-9.82	0.135209	8	14	5	0	1	0	1	28	4	1.6156	0.571	
9	Azoxystrobin	501.57	55.52	459.89	381.78	-8.65	0.181568	22	17	3	5	0	0	3	49	16	2.9055	1.294	4.4
10	bensulide	516.5	77.9	507.25	404.68	-9.56	0.246457	14	24	1	4	0	3	1	47	3.5	2.8663	0.583	3.5
11	bromacil	286.67	45.39	604	487.63	-9.23	0.225529	9	13	2	2	0	0	1	27	4	1.6266	0.692	4.5
12	carbamazepine	317.46	30.07	517.1	404.07	-8.36	0.133609	15	12	2	1	0	0	3	32	11	1.8031	1.25	15
13	carbaryl	278.73	28.46	524.5	408.18	-8.49	0.133653	12	11	1	2	0	0	2	27	8	1.5354	1.091	6
14	carbendiazim	247.34	31.07	481.18	348.92	-9.2	0.150613	9	9	3	2	0	0	2	24	7	1.3568	1	4.5
15	chlorobenzilate	399.67	7.33	329.42	239.54	-9.4	0.027243	16	14	0	3	2	0	2	36	9	2.2577	1.143	5.33
16	chlorothalonil	266.06	28.47	336.17	266.74	-8.61	0.141185	8	0	2	0	4	0	1	14	8	1.5114		
17	chlorpyrifos	399.47	29.21	390.14	301.69	-9.21	0.108197	9	11	1	3	3	1	1	29	3.5	2.1458	0.818	3
18	clofentazine	358.92	22.82	357.29	281.41	-7.74	0.084785	14	8	4	0	2	0	3	30	12	2.0054	1.75	
19	clothianidin	285.06	74.46	696.1	599.68	-9.49	0.34889	6	8	5	2	1	1	1	23	5	1.5727	0.75	3
20	cyanazine	327.06	58.24	545.69	425.9	-9.81	0.247062	9	13	6	0	1	0	1	29	6	1.7698	0.692	
21	cymoxanil	268.43	103.2	758.73	636.58	-8.5	0.471276	7	10	4	3	0	0	0	23	5	1.4517	0.7	2.33
22	cypermethrin	540.57	30.34	380.54	294.67	-9.11	0.085556	22	19	1	3	2	0	3	49	13	2.9623	1.158	7.33
23	cyproconazole	387.64	15.02	407.53	291.89	-10.07	0.059344	15	18	3	1	1	0	3	40	8	2.1543	0.833	15
24	cyromazine	226.99	41.98	419.38	289.47	-10.58	0.22845	6	10	6	0	0	0	2	23	5	1.2036	0.6	
25	desethylatrazine	249.82	41.76	474.41	355.09	-9.92	0.214231	6	10	5	0	1	0	1	22	4	1.3348	0.6	
26	diazepam	366.02	31.83	414.79	330.7	-8.53	0.123799	16	13	2	1	1	0	3	35	11	2.0659	1.231	16
27	diazinon	430.24	55.38	412.01	337.23	-10.11	0.199323	12	21	2	3	0	1	1	40	3.5	2.2996	0.571	4
28	dichlofluanid	355.05	25.85	340.67	282.59	-9.74	0.118496	9	11	2	2	2	2	1	29	4	2.0611	0.818	4.5
29	diflufenican	411.79	37.1	503.46	411.25	-9.18	0.135431	19	11	2	2	0	0	3	41	13	2.5259	1.727	9.5
30	dimethenamid	375.19	26.82	446.24	356.13	-9.76	0.113943	12	18	1	2	1	1	1	35	4	2.0589	0.667	6
31	dinoseb	291.73	21.26	401	305.51	-7.95	0.101156	10	12	2	5	0	0	1	29	6	1.6821	0.833	2
32	diuron	287.6	24.53	476.4	369.99	-9.31	0.118399	9	10	2	1	2	0	1	24	5	1.5947	0.9	9
33	endosulfan	369.05	140.67	776.18	683.88	-7.4	0.664415	9	6	0	3	6	1	3	27	4	2.0774	1.5	3
34	endrin	357.56	9.56	280.39	221.81	-9.91	0.045284	12	8	0	1	6	0	4	30	6	2.0661	1.5	12
35	ethofumesat	375.02	41.12	380.82	299.72	-9.54	0.16576	13	18	0	5	0	1	2	38	5	2.0446	0.722	2.6
36	famoxadone	482.52	36.13	481.58	379.26	-9.03	0.114047	22	18	2	4	0	0	4	49	15	2.7244	1.222	5.5
37	fenthion	371.76	36.76	343.92	262.64	-9.63	0.145205	10	15	0	3	0	2	1	31	3.5	1.9827	0.667	3.33
38	fluazinam	384.15	18.99	314.16	262.77	-7.35	0.079122	13	4	4	4	2	0	2	34	10	2.4776	3.25	3.25
39	flucythrinate	581.79	32.26	388.82	300.6	-9.17	0.085641	26	23	1	4	0	0	3	58	15	3.3272	1.13	6.5
40	flumioxazin	422.1	39.16	389.97	302.88	-7.57	0.137713	19	15	2	4	0	0	4	44	13	2.4269	1.267	4.75
41	fluorouracil	133.44	56.81	590.7	488.19	-9.42	0.459182	4	3	2	2	0	0	1	12	4	0.7873	1.333	2
42	flusilazole	401.79	29.06	433.57	349.34	-9.93	0.110431	16	15	3	0	0	0	3	39	10	2.3057	1.067	
43	fosthiazate	383.56	41.93	537.32	445.94	-9.76	0.169757	9	18	1	4	0	2	1	35	1.5	2.0868	0.5	2.25
44	imiprothrin	454	43.76	467.97	374.3	-9.86	0.151471	17	22	2	4	0	0	2	46	8	2.4546	0.773	4.25
45	ingarol/irgarol	373.42	24.57	468.92	343.36	-9.83	0.095484	11	19	5	0	0	1	2	37	5	1.9693	0.579	
46	isoproturon	327.64	19.67	445.8	330.19	-9.55	0.087758	12	18	2	1	0	0	1	33	5	1.7711	0.667	12
47	metamitron	268.86	44.82	493.72	384.96	-8.31	0.210403	10	10	4	1	0	0	2	26	8	1.4953	1	10
48	metazachlor	368.91	22.15	439.56	356.65	-9.79	0.093338	14	16	3	1	1	0	2	36	8	2.0795	0.875	14
49	methidathion	345.11	75.78	440.54	371.11	-9.75	0.33677	6	11	2	4	0	3	1	27	2.5	1.8859	0.545	1.5
50	metolachlor	405.93	19.9	421.8	331.96	-9.78	0.078728	15	22	1	2	1	0	1	41	5	2.2736	0.682	7.5
51	metoxuron	304.12	25.14	455.41	345.09	-9.36	0.117471	10	13	2	2	1	0	1	28	5	1.6714	0.769	5
52	metribuzin	285.33	200.38	583.42	481.91	-4.87	1	8	14	4	1	0	1	1	28	4	1.6156	0.571	8
53	monouron	269.51	20.3	462.4	349.44	-9.44	0.101846	9	11	2	1	1	0	1	24	5	1.4723	0.818	9
54	napropamide	402.78	22.2	428.75	350.95	-8.47	0.087602	17	21	1	2	0	0	2	42	8	2.2374	0.81	8.5
55	nitrofen	320.11	37.97	376.98	307.83	-8.29	0.161809	12	7	1	3	2	0	2	26	9	1.7959	1.714	4
56	octhilinone	350.25	27.48	469.33	357.33	-9.11	0.111174	11	19	1	1	0	1	1	33	3	1.7804	0.579	11
57	orbencarb	361.64	11.19	341.47	271.95	-9.9	0.043888	12	16	1	1	1	1	1	32	5	1.9572	0.75	12
58	parathion	367.28	69.77	355.89	298.18	-8.4	0.271542	10	14	1	5	0	1	1	32	4.5	1.9934	0.714	2
59	phenmedipham	400.04	35.96	518.15	404.02	-9.58	0.119979	16	16	2	4	0	0	2	39	10	2.2282	1	4
60	procymidone	343.71	27.5	355.28	287.8	-9.92	0.118565	13	11	1	2	2	0	3	31	8	1.855	1.182	6.5
61	propachlor	303.79	25.15	428.18	339.01	-10.41	0.118884	11	14	1	1	0	0	1	27	5.5	1.5094	0.786	11
62	propaquizafop	574.96	34.95	412.59	329.29	-7.8	0.087053	22	22	3	5	1	0	3	55	13	3.1569	1	4.4
63	propioconazole	426.49	22.92	372.52	282.6	-9.93	0.083227	15	17	3	2	2	0	3	41	8	2.3354	0.882	7.5
64	propoxur	306.47	20.1	515.02	397.48	-9.84	0.092388	11	15	1	3	0	0	1	30	5	1.6483	0.733	3.67
65	pymetrozine	289.85	29.4	435.27	311	-8.4	0.125862	10	11	5	1	0	0	2	28	8	1.5951	0.909	10
66	sulfentrazone	386.04	65.93	480.78	386.38	-9.23	0.256318	11	10	4	3	2	1	2	34	7	2.2798	1.1	3.67
67	tebutam	375.48	11.89	377.45	297.14	-10.21	0.050388	15	23	1	1	0	0	1	40	5	2.0925	0.652	15
68	terbutryne	365.87	26.47	452.02	327.62	-9.95	0.101706	10	19	5	0	0	1	1	35	4	1.9375	0.526	
69	thiazopyr	428.16	34.78	384.51	319	-8.57	0.141843	16	17	2	2	0	1	2	44	7	2.5918	0.941	8
70	triclabendazole	404.58	26.34	548.04	440.46	-8.79	0.092262	14	9	2	1	3	1	3	32	10	2.2364	1.556	14
71	triclosan	326.3	6.64	403.66	289.22	-9.36	0.027672	12	7	0	2	3	0	2	25	8	1.8028	1.714	6
72	triflumuron	380.17	53.11	766.22	659.52	-8.79	0.195344	15	10	2	3	1	0	2	35	10	2.2646	1.5	5
73	trifluralin	378.1	11.91	311.97	241.77	-7.46	0.052786	13	16	3	4	0	0	1	39	6	2.2575	0.813	3.25
74	trilate	384.47	1.86	342.52	272.33	-9.79	0.00764	10	16	1	1	3	1	0	31	2	2.1158	0.625	10
75	vinclozolin	333.55	19.54	351.82	278.91	-9.86	0.084761	12	9	1	3	2	0	2	28	8	1.8389	1.333	4
76	zearalenone	419.27	49.18	552.91	437.57	-8.45	0.185242	18	22	0	5	0	0	2	46	8	2.4541	0.818	3.6

Table S.1: Pearson r correlations between each QSAR parameter and the experimentally determined Abraham solute descriptors for the set of 76 pesticides/pharmaceuticals from Tülp et al.[4] QSAR parameters are listed from most to least significant (based on p-value).

S-parameter correlations	Pearson r	p-value	A-parameter correlations	Pearson r	p-value	B-parameter correlations	Pearson r	p-value	E-parameter correlations	Pearson r	p-value
Log P	-0.345029	0.0052363	log Kaw	-0.7552107	8.57781E-10	#bonds	0.76710053	6.47135E-16	B	0.4203919	0.00015624
B	-0.2683495	0.0190879	Net EI	0.5461048	3.35134E-07	CPK Area (Å^2)	0.76465781	9.07298E-16	log Kaw	-0.5075211	0.00027138
log Kow	-0.250059	0.0304871	HBD Count	0.51166095	2.3148E-06	Surf Area(Surface1)(Å^2)	0.76341344	1.07605E-15	DBE	0.39828012	0.00036635
HBA Count	0.23167684	0.0440383	Diff in property max/min	0.48314785	9.84109E-06	CPK Volume (Å^3)	0.75752505	2.37872E-15	Acc. Area (Å^2)	0.37991672	0.00071174
Acc. Polar Area(75) (Å^2)	0.18674158	0.1062592	log Kow	-0.4606721	3.19765E-05	V	0.75679643	2.61995E-15	Acc. Area(Surface1)(Å^2)	0.34155043	0.00253122
#Cl	-0.1706293	0.1405652	Max ElPot (kJ/mol)	0.39902146	0.000356363	Polarizability	0.75352938	4.02348E-15	#Rings	0.32317443	0.00440623
#N	0.14619139	0.2076234	Tautomers	0.37917989	0.000730382	Surf Volume(Surface1)(Å^3)	0.75290541	4.36371E-15	#N	0.28674522	0.01202757
Net EI	0.1458393	0.208732	Property Max(Surface1) kJ	0.35907139	0.001445645	McGowan Vol (cm^3/mol)	0.75097151	5.60351E-15	Surf Area(Surface1)(Å^2)	0.27051972	0.01810313
Energy (au)	0.14114591	0.2239134	Surf Volume(Surface1)(Å^3)	-0.3537479	0.001719603	Acc. Area (Å^2)	0.74117626	1.92175E-14	Acc. Polar Area(75) (Å^2)	0.26193909	0.02226868
#F	-0.14054	0.2259283	Surf Area(Surface1)(Å^2)	-0.3416266	0.002525231	Acc. Area(Surface1)(Å^2)	0.74000015	2.21999E-14	V	0.25877186	0.02400011
log Koc	-0.1671472	0.2270255	Acc. Area(Surface1)(Å^2)	-0.3379065	0.002832813	CPK Ovality	0.71641305	3.43421E-13	CPK Ovality	0.25841975	0.02419948
Diff in property max/min	0.13779977	0.2351985	CPK Ovality	-0.3294308	0.00366201	#C	0.67410185	2.48013E-11	CPK Area (Å^2)	0.25541041	0.02596149
HOMO-LUMO Gap	0.13740007	0.2365724	Acc P-Area/Acc Area	0.32422712	0.004272313	Molecular Wt. (amu)	0.62864621	1.19905E-09	Molecular Wt. (amu)	0.25293461	0.02749145
E	0.1309268	0.2595998	log Koc	-0.3816715	0.00440303	#H	0.58481191	2.90195E-08	Surf Volume(Surface1)(Å^3)	0.25123969	0.02858222
log Kf	-0.2295569	0.2696605	V	-0.3225173	0.004491709	DBE	0.47022029	1.81963E-05	CPK Volume (Å^3)	0.25028136	0.02921495
Polar Area(75) (Å^2)	0.12785278	0.2710505	CPK Area (Å^2)	-0.3186649	0.005023023	E	0.4203919	0.00015624	Polarizability	0.2487269	0.03026637
PSA (Å^2)	0.12549135	0.2800737	McGowan Vol (cm^3/mol)	-0.3115893	0.006145392	#Rings	0.37594862	0.00081749	McGowan Vol (cm^3/mol)	0.24801397	0.03075914
Tautomers	-0.1178849	0.3104842	Polarizability	-0.3104224	0.006350353	Polar Area(75) (Å^2)	0.33246755	0.003342902	Polar Area(75) (Å^2)	0.2478378	0.03088195
log Kaw	-0.1392392	0.3505985	CPK Volume (Å^3)	-0.307755	0.006841637	log Kow	0.27533333	0.016811001	#C	0.21907024	0.05725963
HBD Count	0.09180497	0.4302636	Acc. Area (Å^2)	-0.3062005	0.007143103	S	-0.2683495	0.019087869	PSA (Å^2)	0.18116272	0.11731236
Molecular Wt. (amu)	-0.0912042	0.4332997	Log P	-0.3112181	0.012308417	HBA Count	0.255717	0.025777141	#bonds	0.16955272	0.1431246
#O	0.0875632	0.4519588	Molecular Wt. (amu)	-0.2748217	0.016279339	Acc. Polar Area(75) (Å^2)	0.23238779	0.043375062	log Koc	0.20155952	0.14386617
#S	0.08745973	0.4524955	#bonds	-0.2615985	0.02244962	#O	0.22598313	0.049658109	Tautomers	0.155177	0.18073177
Property Min(Surface1) kJ	-0.0870421	0.454665	E HOMO (eV)	0.26045357	0.02306705	Log P	0.22114807	0.079066188	#S	0.14778684	0.20265203
E LUMO (eV)	-0.0834486	0.47357	PSA (Å^2)	0.24553208	0.03252754	log Kaw	-0.2538174	0.08515172	Energy (au)	-0.1453419	0.21030529
Min ElPot (kJ/mol)	-0.078956	0.497788	Acc. P-Area(Surface1)(Å^2)	0.23950147	0.037182648	Energy (au)	-0.1936268	0.093752965	Min LocIonPot (ev)	-0.1381758	0.23391098
DBE	0.0755661	0.5164802	log Kf	-0.4062572	0.043888541	#F	0.19204784	0.096513448	C/O	0.14264277	0.25322637
E HOMO (eV)	0.07465847	0.5215447	#C	-0.2317737	0.043947451	Min LocIonPot (ev)	-0.1857589	0.108145064	S	0.1309268	0.25959981
Surf Volume(Surface1)(Å^3)	-0.0734624	0.5282566	#H	-0.2284519	0.047152982	E HOMO (eV)	0.18305752	0.113463262	#F	-0.1282637	0.26950057
Surf Area(Surface1)(Å^2)	-0.0732591	0.529402	Polar Area(Surface1)(Å^2)	0.21925605	0.057043576	Acc P-Area/Acc Area	-0.1645504	0.155478028	C/H	0.12750712	0.27564524
McGowan Vol (cm^3/mol)	-0.0724295	0.5340876	Conformers	0.19809981	0.0862687	A	-0.1596941	0.168212758	HBA Count	0.12654188	0.27603515
CPK Area (Å^2)	-0.068914	0.5541683	E LUMO (eV)	0.19140894	0.097648373	#N	0.13154064	0.257353173	Max ElPot (kJ/mol)	0.11792785	0.31030689
Acc. P-Area(Surface1)(Å^2)	0.06813028	0.558694	Energy (au)	0.18189684	0.115809323	E LUMO (eV)	0.1258575	0.278661667	E HOMO (eV)	0.10569396	0.36351039
#H	-0.0666298	0.5674069	#Cl	-0.1776784	0.124651474	PSA (Å^2)	0.10864029	0.350212931	A	0.10520353	0.36575344
V	-0.0663292	0.5691602	#N	0.17195456	0.137462096	Min ElPot (kJ/mol)	-0.1051021	0.3662182	Polar Area(Surface1)(Å^2)	0.10487335	0.36726834
Polar Area(Surface1)(Å^2)	0.06631615	0.5692364	B	-0.1596941	0.168212758	Property Min(Surface1) kJ	-0.1033686	0.37422084	Net EI	0.10466428	0.36822957
CPK Volume (Å^3)	-0.0654546	0.5742755	Acc. Polar Area(75) (Å^2)	0.14683514	0.205607129	Max ElPot (kJ/mol)	-0.096132	0.4087553	Property Max(Surface1) kJ	0.09836844	0.39788941
Conformers	0.06253439	0.5915075	#P	-0.1431381	0.217377658	Property Max(Surface1) kJ	-0.090754	0.435583019	#Cl	0.08969188	0.44099646
Polarizability	-0.0599882	0.6067198	#O	-0.1188405	0.30655076	C/O	0.09292431	0.458024011	Acc. P-Area(Surface1)(Å^2)	0.07915525	0.49670043
#bonds	-0.0588635	0.6134933	#S	-0.1148411	0.323230023	#Cl	-0.0822379	0.480032993	Diff in property max/min	0.07858421	0.49982072
Max ElPot (kJ/mol)	0.0564812	0.6279471	E	0.10520353	0.365753437	Polar Area(Surface1)(Å^2)	0.07109571	0.541663763	HBD Count	0.0705805	0.54460423
CPK Ovality	-0.0541592	0.6421709	HBA Count	-0.1011684	0.384528462	#S	0.06845521	0.556815536	Conformers	0.06848148	0.55666382
#C	-0.0537636	0.6446075	Dipole (debye)	0.09414569	0.418549823	Conformers	0.06789292	0.560068035	E LUMO (eV)	0.06801442	0.55936444
Dipole (debye)	0.04740548	0.6842644	Polar Area(75) (Å^2)	0.09253105	0.426610247	HOMO-LUMO Gap	-0.0543993	0.640694396	Dipole (debye)	-0.0554716	0.63411557
Min LocIonPot (ev)	-0.0453977	0.6969731	HOMO-LUMO Gap	-0.0920097	0.429231744	Dipole (debye)	0.05125732	0.660129456	log Kow	0.04538638	0.69901149
Property Max(Surface1) kJ	0.04253194	0.715257	#F	0.07695499	0.508778865	HBD Count	-0.0445088	0.702626871	Property Min(Surface1) kJ	0.04146764	0.72208905
Acc. Area (Å^2)	-0.0255156	0.8268148	DBE	-0.0764637	0.511496337	log Koc	-0.033346	0.810814848	#O	-0.0408275	0.72620869
Acc. Area(Surface1)(Å^2)	-0.0250619	0.8298475	Min ElPot (kJ/mol)	-0.0616319	0.596879528	#P	0.02541291	0.827500741	Min ElPot (kJ/mol)	0.03991989	0.73206317
#P	0.02101191	0.8570257	Property Min(Surface1) kJ	-0.0470998	0.686193894	Acc. P-Area(Surface1)(Å^2)	0.01522527	0.8961403	log Kf	0.06635238	0.75266723
A	-0.0194934	0.8672605	#Rings	-0.0440674	0.705440121	Net EI	-0.0145091	0.901000291	Acc P-Area/Acc Area	-0.0356973	0.75949657
C/H	0.01395431	0.9054149	C/O	-0.0358196	0.775237275	log Kf	-0.0167038	0.936834865	Log P	-0.0365776	0.77415083
#Rings	0.00981747	0.932921	S	-0.0194934	0.867260535	Diff in property max/min	-0.0081035	0.944611608	HOMO-LUMO Gap	-0.0258993	0.82425159
Acc P-Area/Acc Area	0.00479979	0.967176	C/H	0.01319621	0.910531716	Tautomers	-0.0074936	0.948774777	#H	-0.0199347	0.86428413
C/O	0.00401612	0.974469	Min LocIonPot (ev)	-0.0119889	0.918130872	C/H	-0.0014698	0.990014907	#P	0.00321534	0.97800814

Table S.2: QSAR data obtained in Spartan '18 the neonicotinoids studied in our sorption experiments and the microbial,[11–15] photolysis,[16] chlorination,[17] and hydrolysis[16–18] transformation products previously identified.

	A	B	C	D	E	F	G	H	I	J	K	L	M	N	O	P	Q	R	S	T	U
Imidacloprid	-1233	-8.83	14.29	2	0.35	255.665	12	248.37	221.79	67.082	1.4	178.88	-271.86	245.91	160.41	118.96	11.27	1.47	0	57.2	5
desnitro-imidacloprid	-1028.6	-8.15	7.64	1	0.95	210.668	48	223.77	199.45	40.441	1.36	162.94	-254.15	271.56	124.35	96.22	10.26	1.1	0	55.41	2
Imidacloprid urea	-1048.5	-8.59	5.71	1	0.87	211.652	8	220.84	196.43	35.15	1.35	160.32	-238.21	239.97	122.85	94.34	11.05	0.13	1	55.08	4
clothianidin	-1515.7	-8.88	9.89	4	0.61	249.682	36	237.96	201.43	77.636	1.43	171.84	-279.84	416.26	144.3	102.27	11.48	1.54	0	55.48	6
thiamethoxam	-1668.3	-8.76	12.21	0	0.56	291.719	18	261.7	236.01	64.365	1.42	176.87	-281.97	245.42	155.57	110.15	11.56	2.45	0	58.32	7
thiacloprid	-1463.6	-8.75	9.17	0	0.77	252.729	6	251.53	227.19	29.103	1.4	186.57	-302.19	225.88	159.06	114.55	10.63		0	57.56	4
Acetamiprid	-1066.7	-8.57	6.78	0	0.82	222.679	12	243.33	218.91	28.564	1.39	178.99	-298.32	240.65	160.45	119.93	11.3		0	56.92	3
Dinotefuran	-719.54	-8.91	11.5	4	0.53	202.214	36	226.49	193.08	73.301	1.4	155.91	-288.59	376.08	151.56	109.94	11.48	0.58	0	54.81	4
thiacloprid amide	-1540.1	-8.59	9.62	1	0.87	270.744	18	262.74	238.22	51.676	1.41	187.91	-286.92	230.41	155.42	119.09	10.22	1.01	0	58.47	5
CLO 239a	-1790.7	-8.77	5.35	1	0.84	240.114	108	220.61	190.37	33.272	1.38	163.4	-200.25	279.53	76.52	54.68	11.54	0.9	1	54.55	5
CLO 239b	-1790.7	-8.78	5.28	1	0.65	240.114	108	220.71	190.36	33.465	1.38	162.17	-211.1	268.01	70.69	50.08	11.59	0.9	1	54.59	5
CLO-THX-H 270	-1955.8	-8.93	7.83	2	-0.07	271.084	81	228.58	193.33	76.849	1.41	167.13	-221.15	347.68	117.68	83.68	11.64	1.23	1	54.97	8
dn IMI 245 bottom	-1488.1	-8.38	4.31	0	0.84	245.113	48	240.2	214.47	30.629	1.39	172.47	-218.95	150.94	109.85	81.35	10.69	1.98	0	56.6	2
dn IMI 245 top	-1488.1	-8.1	2.97	1	0.56	245.113	48	237.2	214.69	31.086	1.37	172.02	-205.03	236	91.32	70.33	10.8	1.84	0	56.75	2
dn IMI 279	-1947.7	-8.42	4.21	0	0.59	279.558	24	255.64	229.3	20.743	1.41	183.54	-192.86	172.93	142.41	99.41	11.29	2.72	0	57.85	2
IMI 290	-1692.6	-9.02	8.97	0	0.09	290.11	24	268.14	237.46	60.237	1.45	185.05	-264	239.02	164.33	109.58	11.32	2.54	0	58.49	5
IMI Urea 246	-1508	-8.82	4.12	0	0.81	246.097	24	237.39	211.44	25.582	1.38	170.07	-241.89	169.67	131.21	92.26	11.28	0.88	0	56.26	4
THX-H 237	-1496.3	-8.96	5.33	4	0.22	236.639	108	215.53	178.59	88.194	1.41	161.34	-260.96	417.54	133.75	93.79	11.65	0.49	2	53.7	8
THX-H 248	-1483.7	-8.43	7.62	0	0.84	247.706	18	236.88	211.16	30.336	1.38	168.71	-229.07	192.23	113.97	77.55	11.55	0.79	0	56.32	6
imidacloprid olefin	-1231.8	-8.09	15.26	2	0.55	253.649	6	245.43	217.79	66.492	1.4	182.98	-294.73	304.42	163.99	127.05	11.24	1.77	0	57	5
5-hydroxy imidacloprid	-1308.2	-9.01	13.61	2	0.35	271.664	36	256.65	229.21	84.636	1.42	180.86	-263.82	359.09	167.07	120.16	11.23	1.39	1	57.76	6
clothianidin methyl	-1331.2	-8.59	7.34	2	0.86	205.669	36	207.81	175.82	44.39	1.37	154.3	-250.82	330.54	84.58	53.88	11.24	0.16	2	53.41	5

	V	W	X	Y	Z	AA	AB	AC	AD	AE	AF	AG	AH	AI	AJ	AK	AL	AM	AN	AO	AP	AQ	AR
Imidacloprid	-239.24	209.93	272.7	223.56	143.18	300.29	116.77	0.66503	517.77	449.17	-9.18	9	10	5	2	1	0	2	28	7	1.6788	0.9	4.5
desnitro-imidacloprid	-195.69	198.87	254.57	210.74	61.13	279.85	50.94	0.59052	525.71	394.56	-9.1	9	11	4	0	1	0	2	26	6	1.5046	0.8182	
Imidacloprid urea	-197.24	171.51	249.55	205.47	52.07	271.28	45.67	0.58845	478.18	368.75	-9.46	9	10	3	1	1	0	2	25	6	1.4635	0.9	9
clothianidin	-248.01	351.67	268.54	213.42	96.33	285.06	74.46	0.59515	696.1	599.68	-9.49	6	8	5	2	1	1	1	23	5	1.5727	0.75	3
thiamethoxam	-254.12	206.96	282.2	218.41	127.98	319.59	97.74	0.62277	527.39	461.08	-9.32	8	10	5	3	1	1	2	29	6	1.8036	0.8	2.6667
thiacloprid	-250.67	189.4	285.26	237.43	125.01	318.05	99.03	0.61398	528.07	440.07	-9.52	10	9	4	0	1	1	2	26	8	1.7225	1.1111	
Acetamiprid	-245.03	189.23	273.25	225.35	115.77	303.96	91.01	0.67004	538.97	434.26	-9.39	10	11	4	0	1	0	1	26	7	1.6676	0.9091	
Dinotefuran	-253.21	295.01	254.58	205.99	119.72	271.35	95.24	0.70515	664.67	548.22	-9.44	7	14	4	3	0	0	1	28	3	1.4721	0.5	2.3333
thiacloprid amide	-246.29	192.69	288.93	236.68	112.03	327.66	88.55	0.37413	517.33	438.98	-9.46	10	11	4	1	1	1	2	29	7	1.8242	0.9091	10
CLO 239a	-160.7	208.67	251.99	205.05	41.43	273.21	28.16	0.13733	479.78	369.37	-9.61	6	7	3	1	2	1	1	20	4	1.4798	0.8571	6
CLO 239b	-170.17	198.13	248.45	203.66	36.7	272.01	27.12	0.13316	479.11	368.3	-9.43	6	7	3	1	2	1	1	20	4	1.4798	0.8571	6
CLO-THX-H 270	-192.27	264.64	252.56	201.34	78.14	270.06	58.76	0.29184	568.83	456.91	-8.86	5	4	4	3	2	1	1	19	5	1.5136	1.25	1.6667
dn IMI 245 bottom	-166.60	118.2	273	219.88	46.14	303.89	37.64	0.17118	369.89	284.8	-9.22	9	10	4	0	2	0	2	26	6	1.627	0.9	
dn IMI 245 top	-164.73	162.91	268.83	214.79	27.55	302.79	20.46	0.09526	441.03	327.64	-8.66	9	10	4	0	2	0	2	26	6	1.627	0.9	
dn IMI 279	-154.46	141.78	288.69	231.18	78.03	323.68	63.71	0.27559	365.79	296.24	-9.01	9	9	4	0	3	0	2	26	6	1.7494	1	
IMI 290	-236.97	204.35	292.8	228.72	127.34	324.53	92.24	0.40329	503.02	441.32	-9.11	9	9	5	2	2	0	2	28	7	1.8012	1	4.5
IMI Urea 246	-202.64	135.51	268.53	215.03	78.34	295.05	58.17	0.27052	411.56	338.15	-9.63	9	9	3	1	2	0	2	25	6	1.5859	1	9
THX-H 237	-227.90	348.82	244.95	197.56	90.41	251.51	63.09	0.31935	678.5	576.72	-9.18	5	5	4	3	1	1	1	19	5	1.3912	1	1.6667
THX-H 248	-188.49	152.03	263.44	214.05	63.96	293.07	44.88	0.20967	421.3	340.52	-9.27	8	10	3	2	1	1	2	26	5	1.5883	0.8	4
imidacloprid olefin	-260.81	257.88	267.34	223.52	142.71	291.24	117.79	0.52698	599.15	518.69	-8.64	9	8	5	2	1	0	2	26	8	1.6358	1.125	4.5
5-hydroxy imidacloprid	-231.03	279.5	275.99	223.22	142.73	305.12	110.91	0.49686	622.91	510.53	-9.36	9	10	5	3	1	0	2	29	7	1.7375	0.9	3
clothianidin methyl	-207.07	267.4	238.79	197.1	63.71	254.15	42.17	0.21395	581.36	474.47	-9.45	6	8	3	1	1	1		19	4	1.423	0.75	6

Model Development with log K_{ow} as a quality assurance check. Cross-checking the modeled results against literature-reported experimental data in simplified systems is a standard quality assurance step in model construction.[19,20] Due to the absence of experimentally determined solute parameters, we constructed the pp-LFER-inspired QSAR model for a set of 76 pesticides/pharmaceuticals[4] where we replaced experimentally-determined Abraham solute descriptors (E, S, A, B) with correlated/relevant, computationally-derived, QSAR parameters (approach A, **Tables S.10-S.11**). This set of 76 pesticides/pharmaceuticals[4] was chosen assuming it contained compounds with more similar/relatable structure-function activities to neonicotinoids compared to a broader/more diverse set and because clothianidin was included in the 76 pesticides/pharmaceuticals. Literature log K_{ow} values for neonicotinoids were from the EPA CompTox chemistry dashboard;[21,22] experimentally determined values were used, except for the neonicotinoid transformation products (desnitro-imidacloprid, imidacloprid urea, and thiacloprid amide) for which only database-predicted log K_{ow} values were available (**Table S.3**).[22]

The pp-LFER-inspired QSAR-MLR model (approach A) yielded a RMSE of 0.78 log-units, which was only 0.06 log-units higher than when using the traditional pp-LFER previously reported for this data set[4] (**Table S.13A** and **Figure S.4A**). Although not identical to the traditional pp-LFER, this QSAR-MLR model is a close approximation because it is based on the QSAR parameters most-highly correlated with each experimentally-determined solute parameter and has an RMSE within 0.1 log units of the regression obtained for this set of pesticides/pharmaceuticals when using the actual pp-LFER.[4] Nevertheless, when using the pp-LFER-inspired model (approach A), neonicotinoid log K_{ow} (with 95% confidence intervals) was still overpredicted by 0.4-1.3 log units (**Figure S.4A,** QSAR values in **Table S.12**). This exercise highlights that our surrogate pp-LFER model performs similarly to the pp-LFER model provided by Tulp et al.,[4] enabling us to make direct comparisons between the traditional pp-LFER model and the model we developed.

To improve upon the pp-LFER-inspired model (approach A), we constructed a MLR model tailored to the log K_{ow} for the 76 pesticides/pharmaceuticals using the QSAR parameters most important/relevant to the octanol-water partitioning of these pesticides/pharmaceuticals (approach B, **Table S.14**). Using the tailored model (approach B), we obtained an RMSE of 0.6950 (0.03 log-units lower than previously reported with the pp-LFER,[4] **Table S.13B** and **Figure S.4B**). The tailored approach continued to overpredict neonicotinoid log K_{ow} (by 0.5-2.0 log-units) with the exception of clothianidin (note that clothianidin was included in the set of 76 pesticides/pharmaceuticals) (**Figure S.4B**). Note that two neonicotinoids (acetamiprid and thiacloprid) could not be calculated with this model as Spartan could not converge on a Log P value.

Although the traditional and tailored QSAR-MLR models generated using the set of 76 pesticides/pharmaceuticals (approach A and B respectively, **Tables S.13A** and **B**) overpredicted neonicotinoid log K_{ow}, the parameters used within those models can be fit to neonicotinoids specifically. By training the model parameters with neonicotinoids, the coefficients are adjusted to better reflect neonicotinoid partitioning. Thus, with the model parameters from the traditional pp-LFER-inspired QSAR-MLR (approach A) when applied to neonicotinoids specifically, neonicotinoid log K_{ow} predictions (**Figure S.4C**) followed a similar pattern observed with the SMILES-based pp-LFER predictions (**Figure S.5**), wherein lower literature log K_{ow} values were the most overpredicted. The parameters used in the tailored QSAR-MLR (approach B, **Table S.13B**) provided more accurate modeling of neonicotinoid log K_{ow} (predicted log K_{ow} within 0.1

log-units of literature values, **Figure S.4D**), suggesting that these QSAR parameters are more reflective of neonicotinoid log K_{ow} than those in the more traditional pp-LFER-inspired model (approach A). Comparisons between the parameter coefficients for the tailored model for the set of 76 pesticides/pharmaceuticals to that for the neonicotinoid model (**Table S.15**) indicate that the use of a larger suite of pesticides/pharmaceuticals under-predicts the impact of the number of H atoms present and overpredicts in the impact of McGowan volume and Log P. Note, although log P is a water/octanol partition coefficient calculated using the Ghose-Crippen method, the correlation is not strong (pearson r= 0.429, **Figure S.6**), and is used in this instance as a parameter that can be adjusted with other parameters to more accurately predict neonicotinoid log Kow.

Using the tailored approach, we modeled octanol-water partitioning for neonicotinoids (correlations in **Table S.16**), generating a model with an RMSE of 0.01723 that can accurately predict acetamiprid and thiacloprid amide log K_{ow} during external validation (**Table S.13C**, **Figure S.4E**). It is important to note that transformation products did not have experimentally determined log K_{ow} values reported and are modeled here with EPA CompTox predicted values, thus potentially introducing additional error within the model and predictions made therein. This model demonstrates that tailoring the MLR to neonicotinoids allows us to focus on the most important/relevant QSAR parameters for describing neonicotinoid partitioning, leading to more accurate predictions than can be generated with the more traditional pp-LFER. Multiple QSAR parameters for the neonicotinoid log K_{ow} model relate to energy terms (e.g., minimum local ionization potential, total energy, and polarizability, **Table S.13C**), which are largely overlooked in both the traditional pp-LFER and when using a diverse set of structurally dissimilar contaminants, and may be generalizable in developing predictive tools for polar organic contaminants.

Table S.13: Multiple linear regression model parameters and internal validation information for three different models (A) traditional approach relating Spartan QSAR descriptors to experimentally determined Abraham solute parameters reported in Tülp et al.[4] for modeling pesticide log K_{ow}. (B) Tailored approach relating literature log K_{ow} values to Spartan QSAR descriptors for the 76 pesticides/pharmaceuticals in Tülp et al.[4] (C) Tailored approach relating log K_{ow} for neonicotinoids to Spartan QSAR descriptors. This model excluded acetamiprid and thiacloprid amide, which were used for external validation.

(A)

Descriptor	Coefficient	Standard Error	t-test	p-value	VIF
Intercept	-5.553	1.885	2.945	0.0044	
E: #N	-0.2418	0.06536	3.699	0.0004	1.129
S: Acc. Polar Area(75) (Å²)	-0.01099	0.004537	2.421	0.0181	1.415
A: E HOMO (eV)	-0.7184	0.2163	3.322	0.0014	1.234
B: HBA count	-0.1325	0.06442	2.057	0.0435	1.470
V: McGowan Vol (cm^3/mol)	2.173	0.2037	10.66	<0.0001	1.263
R^2= 0.7015, R_{adj}^2= 0.6799, RMSE= 0.7793, F=32.43, p<0.0001, n=75					

(B)

Descriptor	Coefficient	Standard Error	t-test	p-value	VIF
Intercept	1.679	0.6071	2.766	0.0076	
#H	-0.06021	0.02498	2.410	0.0192	2.234
McGowan Vol (cm^3/mol)	1.810	0.2419	7.483	<0.0001	2.046
#N	-0.3382	0.06595	5.129	<0.0001	1.272
Diff in property max/min	-0.003267	0.001327	2.463	0.0168	1.238
Log P	0.3580	0.08214	4.359	<0.0001	1.406
R^2= 0.7743, R_{adj}^2= 0.7545, RMSE= 0.6950, F=39.11, p<0.0001, n=63					

(C)

Descriptor	Coefficient	Standard Error	t-test	p-value	VIF
Intercept	25.06	1.195	20.98	0.0303	
Min LocIonPot (ev)	0.4624	0.06425	7.197	0.0879	3.380
Energy (au)	-0.001648	0.0001161	14.20	0.0447	5.004
Acc. Area (Å²)	0.06453	0.003729	17.31	0.0367	5.812
#C	0.4603	0.02818	16.34	0.0389	5.094
Polarizability	-0.8295	0.03362	24.68	0.0258	7.387
R^2= 0.9992, R_{adj}^2= 0.9955, RMSE=0.01723, F=263.8, p=0.0467, n=7					

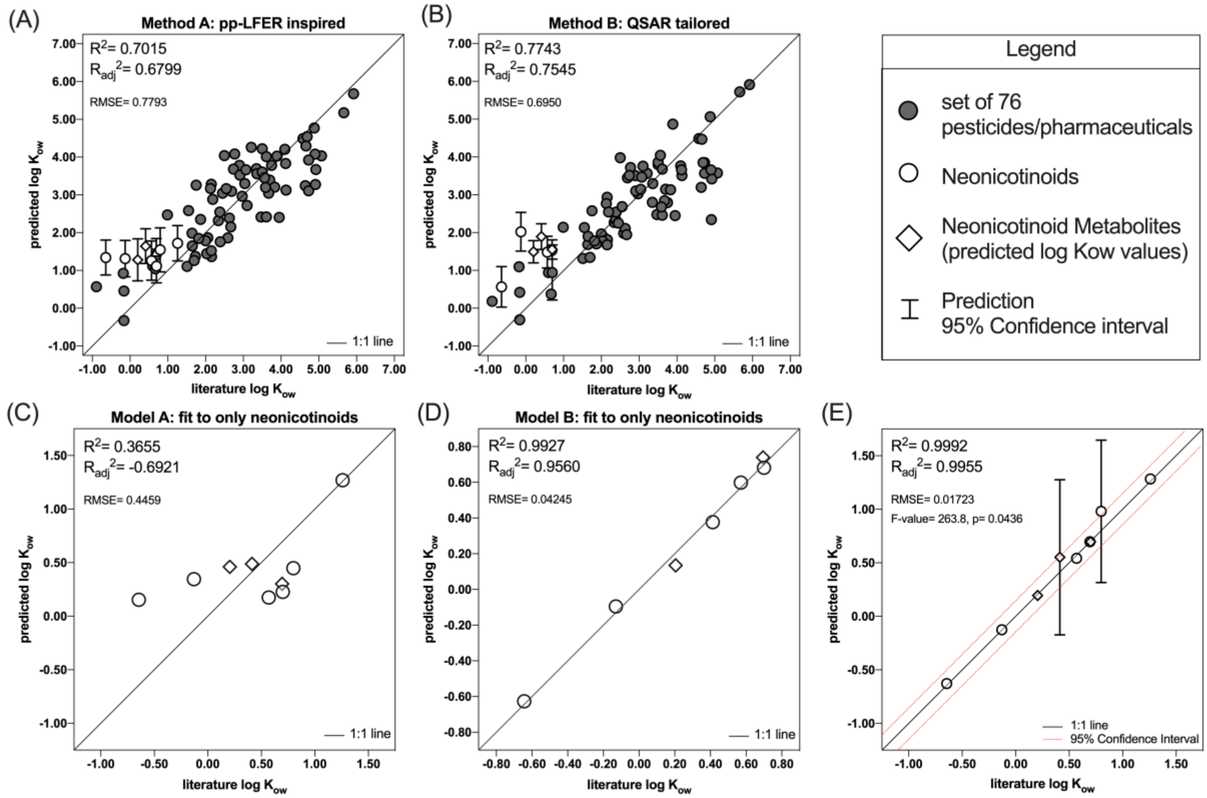

Figure S.4: (A) Predicted vs. literature log K_{ow} values using approach A based on QSAR parameters most-correlated to experimentally determined Abraham solute parameters for the 76 pesticides/pharmaceuticals.[4] (B) Predicted vs. literature log K_{ow} values using approach B based on QSAR parameters most correlated with pesticide/pharmaceutical log K_{ow} (for the 76 pesticides/pharmaceuticals).[4] (C) Multiple linear regression of neonicotinoids when using the parameters from panel A (approach A). (D) Multiple linear regression of neonicotinoids when using the parameters from panel B (approach B). (E) Multiple linear regression tailoring QSAR descriptors to neonicotinoid log K_{ow}. Error bars represent the 95% confidence intervals of predicted log K_{ow} for compounds used for external validation.

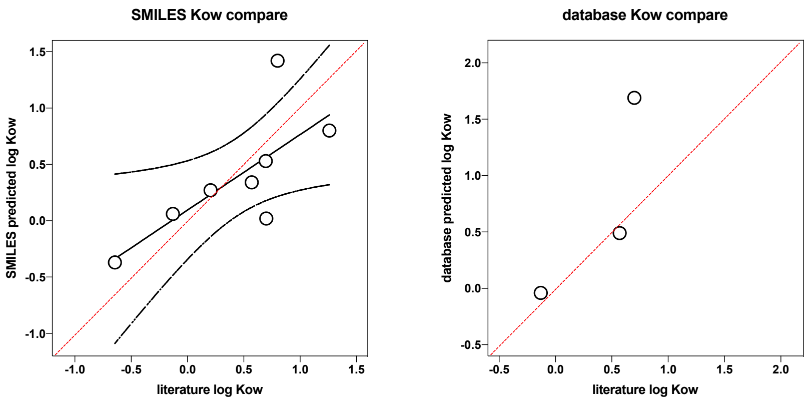

Figure S.5: Predicted neonicotinoid log Kow partitioning using UFZ-LSER SMILES predicted solute parameters (left) and experimentally determined (clothianidin) or Abraham database provided (imidacloprid and thiamethoxam) solute parameters (right). The linear relationship and dashed 95% confidence intervals on the SMILES predicted figure highlights the inexact predictability for the pp-LFER/ SMILES to predict neonicotinoid log Kow.

Table S.3: Pearson r correlations between each QSAR parameter and the literature log K_{ow} values for the 76 pesticides/pharmaceuticals from Tülp et al.[4] Parameters are listed from most to least significant (based on p-value).

pesticide/pharmaceutical QSAR-logK_{ow} correlations	Pearson r	p-value
McGowan Vol (cm^3/mol)	0.72219821	2.62021E-13
Molecular Wt. (amu)	0.71472261	5.95121E-13
Surf Volume(Surface1)(Å³)	0.71448029	6.10892E-13
Polarizability	0.71117517	8.70429E-13
CPK Volume (Å³)	0.71070253	9.15267E-13
Surf Area(Surface1)(Å²)	0.7041252	1.82256E-12
CPK Area (Å²)	0.70046558	2.65225E-12
Acc. Area (Å²)	0.68463296	1.26251E-11
Acc. Area(Surface1)(Å²)	0.66538372	7.41219E-11
#C	0.63807734	7.38809E-10
CPK Ovality	0.62805016	1.62504E-09
#bonds	0.60891918	6.77831E-09
Energy (au)	-0.4966415	5.84611E-06
#N	-0.4556789	3.98911E-05
Acc P-Area/Acc Area	-0.4279354	0.000128279
Log P	0.41495652	0.000720897
#H	0.38025902	0.000764905
DBE	0.365762	0.00125101
#Rings	0.34970667	0.002102122
Tautomers	-0.3457802	0.002377053
Net EI	-0.3394704	0.002886806
#Cl	0.32795041	0.004074634
Diff in property max/min	-0.2896018	0.01173026
Acc. P-Area(Surface1)(Å²)	-0.2672268	0.020465471
PSA (Å²)	-0.2649008	0.021631721
#O	0.25744133	0.025761434
C/H	0.25654685	0.027356231
Max ElPot (kJ/mol)	-0.2515406	0.029484284
#F	0.24842805	0.031624166
Property Max(Surface1) kJ	-0.2252412	0.05202469
Polar Area(Surface1)(Å²)	-0.2082312	0.073012333
#P	0.14268327	0.222018433
Conformers	0.11025862	0.346343656
#S	0.09246957	0.430081457
C/O	0.07191406	0.569167664
Min LocIonPot (ev)	-0.0664458	0.571122627
HBA Count	-0.0609666	0.603341282
Acc. Polar Area(75) (Å²)	-0.0608676	0.603930343
HOMO-LUMO Gap	0.05999976	0.609108237
HBD Count	-0.0568508	0.628055947
Polar Area(75) (Å²)	0.04839427	0.680112897
E LUMO (eV)	-0.0380536	0.74583521
Min ElPot (kJ/mol)	0.03464449	0.767933265
E HOMO (eV)	0.029213	0.803518723
Dipole (debye)	-0.0213129	0.855978473
Property Min(Surface1) kJ	0.01634123	0.889330419

Table S.4: QSAR descriptors from approach B for modeling the set of 76 pesticides/pharmaceuticals) fit specifically to neonicotinoid literature log Kow values. Two neonicotinoids (thiacloprid and acetamiprid) were omitted from this model as Spartan was unable to calculate their Log P values. Although this particular multiple linear regression can accurately predict neonicotinoid log Kow, the model does not follow OECD requirements for QSAR modeling. It is important to note that although Log P (a water/octanol partition coefficient calculated by the Ghose-Crippen method) is included as a model parameter in modeling log K_{ow}, we know that Log P provided by Spartan does not strongly correlate to literature log K_{ow} values for the pesticides and pharmaceuticals analyzed by Tulp et al. (see **Figure S.6** below). The use of log P in this model can be considered as a baseline by which log Kow can be predicted with the help of other model parameters (e.g., #H, #N, McGowan volume, and difference in minimum and maximum electron ionization potential).

Descriptor	Coefficient	Standard Error	t-test	p-value	VIF
Intercept	1.778	0.8024	2.217	0.2698	
#H	-0.2728	0.02622	10.41	0.0610	1.254
McGowan Vol (cm^3/mol)	0.8293	0.3956	2.096	0.2834	2.038
#N	0.4533	0.1667	2.719	0.2244	8.819
Diff in property max/min	-0.002120	0.0007896	2.685	0.2269	2.320
Log P	-0.7879	0.1570	5.020	0.1252	7.576

R^2= 0.9927, R_{adj}^2= 0.9560, RMSE= 0.04245, F=27.08, p=0.1448, n=7

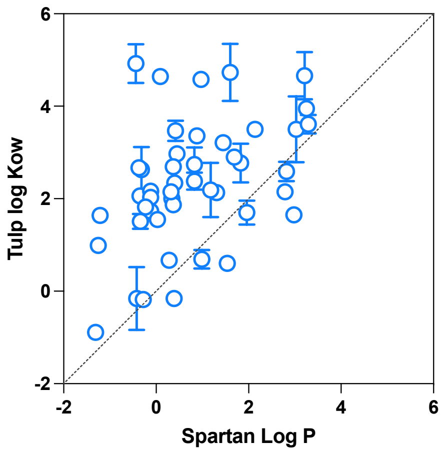

Figure S.6: Spartan calculated log P vs literature log Kow for the 46 Tulp compounds that a Log P value could be calculated for by Spartan. The Pearson r correlation between literature log K_{ow} and Spartan calculated Log P was 0.429, p=0.003, n=46.

Table S.5: Pearson r correlations between each QSAR parameter and the literature log K_{ow} values for neonicotinoids and metabolites (as obtained from the EPA CompTox database). Parameters are listed from most to least significant (based on p-value).

neonicotinoid QSAR log K_{ow} correlations	Pearson r	p-value
C/H	0.88412305	0.00355961
DBE	0.80885792	0.01505146
#H	-0.7464627	0.03338943
#Cl	0.72660922	0.04118294
#O	-0.7037104	0.05143264
Acc. Area (Å²)	0.64677487	0.08305176
Acc. Area(Surface2)(Å²)	0.64184584	0.08621312
PSA (Å²)	-0.5819052	0.1302088
#bonds	-0.5748303	0.13608373
Conformers	-0.5602769	0.14862301
#C	0.52436065	0.18217709
Acc. P area/Acc.Area	-0.4485753	0.26493811
C/O	0.59613486	0.28870108
Tautomers	-0.4124397	0.30989843
Max ElPot (kJ/mol)	-0.3982655	0.3284585
Energy (au)	-0.3893892	0.34033916
Dipole (debye)	-0.389346	0.3403974
Surf Area(Surface2)(Å²)	0.38737026	0.34306881
Surf Volume(Surface2)(Å³)	0.35719959	0.38503968
Min LocIonPot (ev)	-0.3517594	0.39283765
EI pot diff	-0.3452791	0.40221555
E LUMO (eV)	0.32907568	0.4260808
Property Max(Surface2) kJ	-0.3199432	0.43978756
Property max-min	-0.2952818	0.47768727
CPK Volume (Å³)	0.28512249	0.49366243
Polarizability	0.27788966	0.50515968
HOMO/LUMO	-0.275576	0.50885877
McGowan Vol (cm^3/mol)	0.26300108	0.52914069
#S	0.24633906	0.55645981
#Rings	0.202976	0.62974387
CPK Area (Å²)	0.20186173	0.63166543
HBD Count	0.17760058	0.67393499
Molecular Wt. (amu)	0.1544576	0.71496519
#N	-0.1414727	0.73825681
HBA Count	-0.1383505	0.74388405
Acc. Polar Area(75) (Å²)	0.13595494	0.74820826
Property Min(Surface2) kJ	0.10340367	0.80749572
E HOMO (eV)	0.10045906	0.81290272
CPK Ovality	-0.0960346	0.82103918
Min ElPot (kJ/mol)	-0.0930723	0.82649454
Polar Area(75) (Å²)	0.07851268	0.85339258
Polar Area(Surface2)(Å²)	-0.0672965	0.87419959
Log P	-0.0695636	0.89582288
Acc. P-Area(Surface2)(Å²)	-0.0519365	0.90279404

Table S.6: Pearson r correlations between each QSAR parameter and the experimentally determined log K_F values associated with neonicotinoid/metabolite sorption to nonfunctionalized (nF-CNTs) and oxidized carbon nanotubes (O-CNTs). Parameters are listed from most to least significant (based on p-value).

nF-CNT and O-CNTcombined log K_F correlations	Pearson r	p-value
Acc. Area(Surface2)(Å²)	0.84185957	4.3191E-05
Surf Area(Surface2)(Å²)	0.83026855	6.8595E-05
Acc. Area (Å²)	0.82935931	7.1027E-05
Surf Volume(Surface2)(Å³)	0.77428875	0.00043009
Min ElPot (kJ/mol)	-0.7719737	0.00045886
Log P	0.84022721	0.00062297
HBD Count	-0.7344822	0.00119416
McGowan Vol (cm^3/mol)	0.71765624	0.00174718
CPK Area (Å²)	0.69917316	0.00257805
Polar Area(75) (Å²)	0.66770796	0.00470681
CPK Volume (Å³)	0.66318249	0.00510396
Polarizability	0.66090709	0.00531354
Acc. Polar Area(75) (Å²)	0.62979424	0.00893384
C/O	-0.7366565	0.015094
Polar Area(Surface2)(Å²)	0.57223207	0.02054081
Property Min(Surface2) kJ	-0.5699182	0.02117625
CPK Ovality	0.53801384	0.03157672
Acc. P-Area(Surface2)(Å²)	0.53698992	0.03196494
DBE	0.53656101	0.03212863
Energy (au)	-0.5208437	0.03857862
#S	0.51629062	0.04061749
Molecular Wt. (amu)	0.49497754	0.0512548
#N	0.49200751	0.05288693
C/H	0.45393286	0.07736786
#H	-0.3627004	0.16737883
#Cl	0.33110015	0.21032519
Tautomers	-0.3298132	0.21221097
#O	-0.3055207	0.24984296
#C	0.26530572	0.32066795
Dipole (debye)	0.18734337	0.4871954
Property max-min	0.18232222	0.4991484
PSA (Å²)	-0.1614707	0.55021867
Max ElPot (kJ/mol)	-0.1548705	0.56684458
Conformers	-0.1506957	0.57746988
#Rings	-0.0961082	0.72328667
Min LocIonPot (ev)	-0.0914701	0.73618614
E HOMO (eV)	-0.0810191	0.76549057
HBA Count	0.07785924	0.77441194
Acc. P area/Acc.Area	0.07401027	0.78531451
EI pot diff	0.07311709	0.78784994
E LUMO (eV)	-0.0664838	0.8067407
HOMO/LUMO	-0.0432008	0.87378019
#bonds	0.00963586	0.97174697
Property Max(Surface2) kJ	1.8414E-05	0.999946

Matlab Sorption Predictions. Sigmund et al.[23] recently published a deep learning neural network approach to predict the Freundlich sorption constant (log K_F) and n that is applicable to ionizable and polar organic contaminants. Using their available freely available MatLab interface (available at pubs.acs.org/doi/abs/10.1021/acs.est.9b06287) we predicted the log K_F for imidacloprid, clothianidin, thiamethoxam, thiacloprid, acetamiprid, dinotefuran, imidacloprid urea, and desnitro-imidacloprid sorption to the F200 GAC, nf-CNTs, O-CNTs, and N-CNTs used in this study. Information regarding sorbent surface characteristics (% carbon content, H/C, O/C, specific surface area [SSA, m^2 g^{-1}]) were estimated. Our Calgon F200 GAC surface chemistry was assumed to be similar to that in a previous study by Selmi et al. who reported surface characteristics for Calgon F200 GAC (which reported ~86.21% C, ~0.51% H, ~5.49% O, ~0.37% N, and C/H ratio of 169).[24] Surface characterizations for carbon nanotubes were assumed based on vendor specifications: nF-CNTs (~98% C, ~0.01% H, ~2% O), O-CNTs (~94% C, ~0.5% H, ~5% O), and N-CNTs (~92% C, ~0.7% H, ~1% O, ~6% N). A pH=7 was used for all sorption isotherms. Neonicotinoid-specific information (log D_{ow}, percent negative species, and percent positive species) were obtained from the chemicalize dashboard (https://chemicalize.com/app).

Table S.7: Input information for deep-learning Matlab predictions for neonicotinoid/metabolite sorption to F200 GAC, nF-CNTs, O-CNTs, and N-CNTs.

Sorbent	Neonicotinoid	Carbon content (%)	H/C	O/C	SSA m^2/g	pH
GAC	imidacloprid	86.21	0.006	0.064	667.19	7
nF-CNTs	imidacloprid	98	0.0001	0.02	192.83	7
O-CNTs	imidacloprid	94	0.005	0.05	122.38	7
N-CNTs	imidacloprid	92	0.008	0.011	218.21	7
GAC	clothianidin	86.21	0.006	0.064	667.19	7
nF-CNTs	clothianidin	98	0.0001	0.02	192.83	7
O-CNTs	clothianidin	94	0.005	0.05	122.38	7
N-CNTs	clothianidin	92	0.008	0.011	218.21	7
GAC	thiamethoxam	86.21	0.006	0.064	667.19	7
nF-CNTs	thiamethoxam	98	0.0001	0.02	192.83	7
O-CNTs	thiamethoxam	94	0.005	0.05	122.38	7
N-CNTs	thiamethoxam	92	0.008	0.011	218.21	7
GAC	thiacloprid	86.21	0.006	0.064	667.19	7
nF-CNTs	thiacloprid	98	0.0001	0.02	192.83	7
O-CNTs	thiacloprid	94	0.005	0.05	122.38	7
N-CNTs	thiacloprid	92	0.008	0.011	218.21	7
GAC	acetamiprid	86.21	0.006	0.064	667.19	7
nF-CNTs	acetamiprid	98	0.0001	0.02	192.83	7
O-CNTs	acetamiprid	94	0.005	0.05	122.38	7
N-CNTs	acetamiprid	92	0.008	0.011	218.21	7
GAC	dinotefuran	86.21	0.006	0.064	667.19	7
nF-CNTs	dinotefuran	98	0.0001	0.02	192.83	7
O-CNTs	dinotefuran	94	0.005	0.05	122.38	7
N-CNTs	dinotefuran	92	0.008	0.011	218.21	7
GAC	imidacloprid urea	86.21	0.006	0.064	667.19	7
nF-CNTs	imidacloprid urea	98	0.0001	0.02	192.83	7
O-CNTs	imidacloprid urea	94	0.005	0.05	122.38	7
N-CNTs	imidacloprid urea	92	0.008	0.011	218.21	7
GAC	desnitro imidaclo	86.21	0.006	0.064	667.19	7
nF-CNTs	desnitro imidaclo	98	0.0001	0.02	192.83	7
O-CNTs	desnitro imidaclo	94	0.005	0.05	122.38	7
N-CNTs	desnitro imidaclo	92	0.008	0.011	218.21	7

Table S.19: Rank order of Freundlich constants (log K_F) determined experimentally and predicted with the Matlab interface developed by Sigmund et al.[23]

Descending order of sorption	GAC experimental	GAC Deep-learning predicted	nF-CNTs experimental	nF-CNTs Deep-learning predicted	O-CNTs experimental	O-CNTs Deep-learning predicted	N-CNTs experimental	N-CNTs Deep-learning predicted
1.	imidacloprid	thiamethoxam	thiacloprid	thiamethoxam	thiacloprid	thiacloprid	thiacloprid	thiamethoxam
2.	acetamiprid	thiacloprid	acetamiprid	thiacloprid	imidacloprid	thiamethoxam	imidacloprid	acetamiprid
3.	clothianidin	acetamiprid	clothianidin	acetamiprid	clothianidin	acetamiprid	acetamiprid	thiacloprid
4.	thiacloprid	imidacloprid	imidacloprid	imidacloprid	acetamiprid	imidacloprid	clothianidin	imidacloprid
5.	dinotefuran	clothianidin	thiamethoxam	dinotefuran	thiamethoxam	clothianidin	thiamethoxam	clothianidin
6.	imidacloprid urea	imidacloprid urea	dinotefuran	clothianidin	desnitro-imidacloprid	dinotefuran	dinotefuran	dinotefuran
7.	thiamethoxam	dinotefuran	imidacloprid urea	desnitro-imidacloprid	dinotefuran	imidacloprid urea	imidacloprid urea	IMI Urea
8.	desnitro-imidacloprid	desnitro-imidacloprid	desnitro-imidacloprid	imidacloprid urea	imidacloprid urea	desnitro-imidacloprid	desnitro-imidacloprid	desnitro-imidacloprid

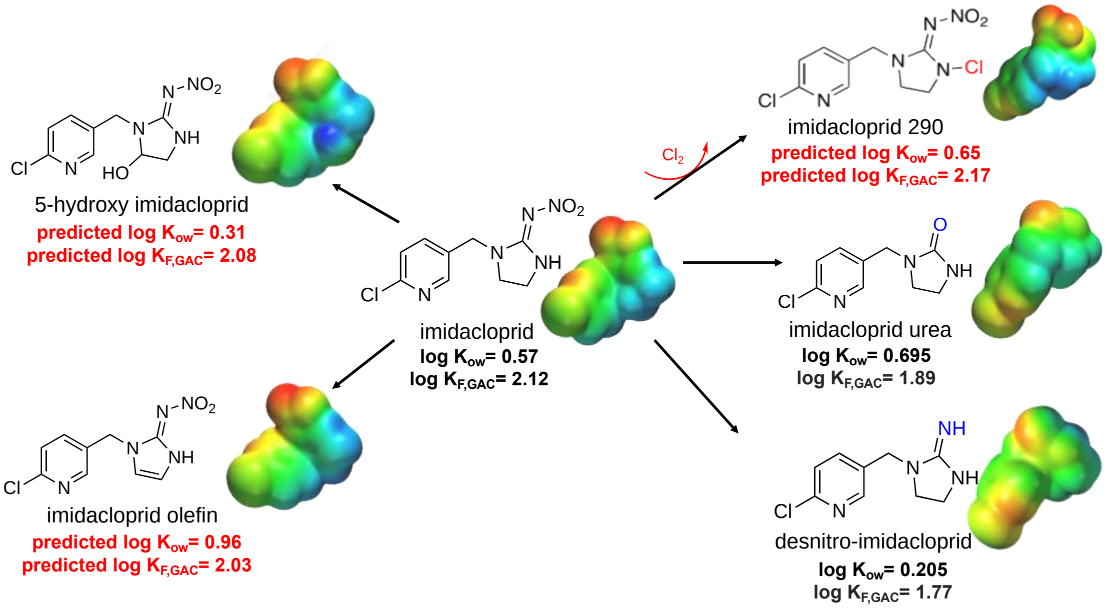

Figure S.7: Electron ionization energy diagrams for imidacloprid and the five imidacloprid transformation products studied (desnitro-imidacloprid and imidacloprid urea) and predicted (5-hydroxy imidacloprid, imidacloprid olefin, and the chlorination product imidacloprid 290). Arrows show parent to product relationships (e.g., imidacloprid is degraded to desnitro imidacloprid which is degraded to imidacloprid urea). Electron ionization energy diagrams range from -250 (red) to 250 (blue) kJ and plot with an iso val of 99.8% 0.0006 e-/au^3.

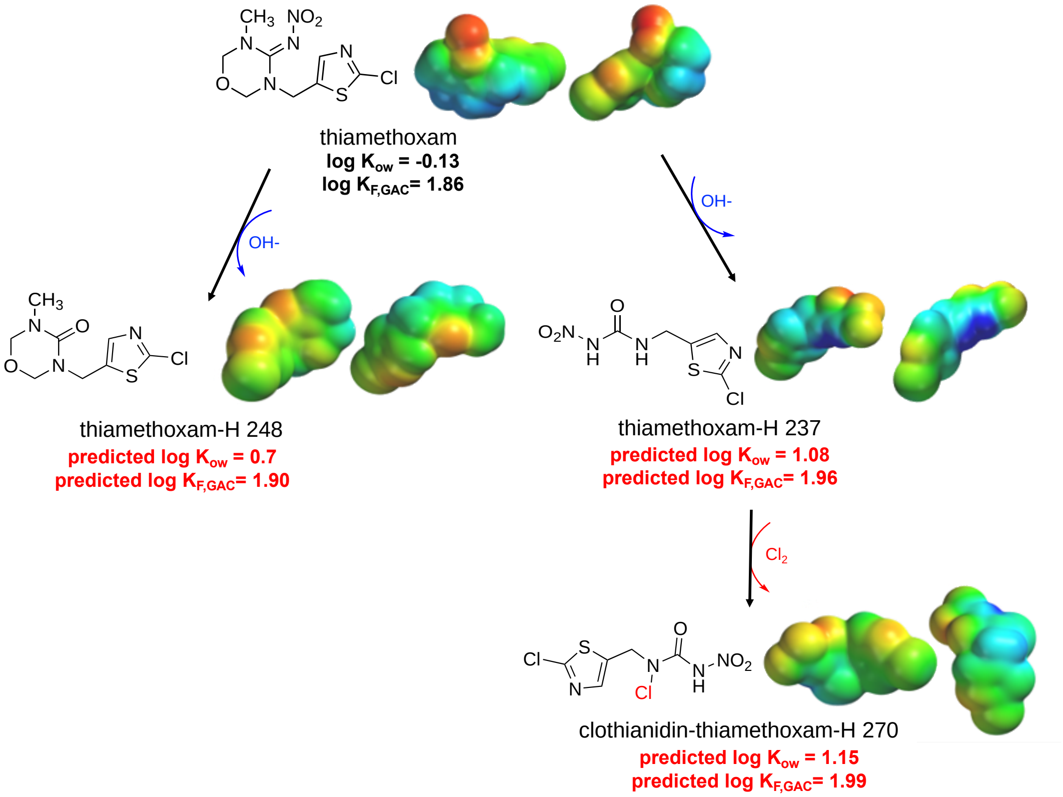

Figure S.8: Electron ionization energy diagrams thiamethoxam, the hydrolysis products thiamethoxam-248 and thiamethoxam 237, and chlorination product clothianidin-thiamethoxam-H 270. Electron ionization energy diagrams range from -250 (red) to 250 (blue) kJ and plot with an iso val of 99.8% 0.0006 e-/au^3.

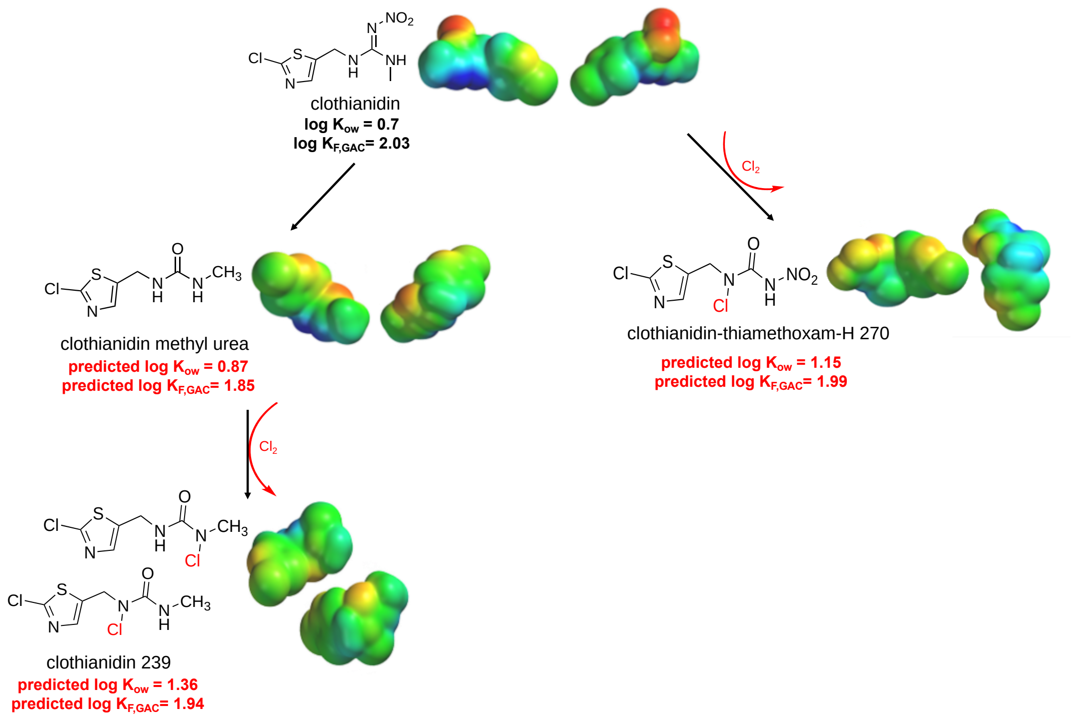

Figure S.9: Electron ionization energy diagrams for clothianidin, the transformation product clothianidin methyl urea, and chlorination products clothianidin 239 and clothianidin-thiamethoxam-H 270. Electron ionization energy diagrams range from -250 (red) to 250 (blue) kJ and plot with an iso val of 99.8% 0.0006 e-/au^3.

(A)

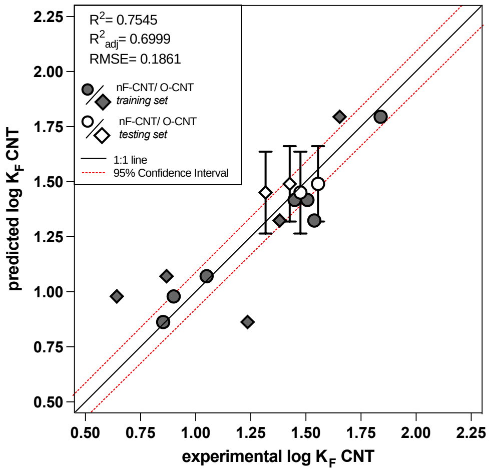

(B)

Descriptor	Coefficient	Standard Error	t-test	p-value	VIF
Intercept	-2.179	0.6690	3.256	0.0099	
Property Min(Surface2) kJ	-0.01041	0.002451	4.249	0.0021	1.029
C/H	1.228	0.3264	3.763	0.0045	1.029
R^2= 0.7545, R_{adj}^2= 0.6999, RMSE=0.1861, n=14					

(C)

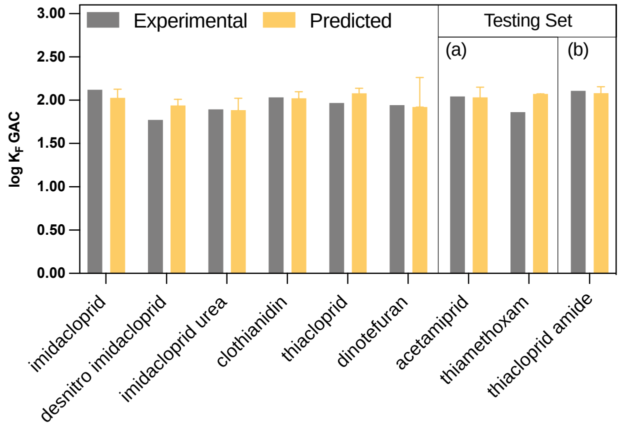

Figure S.10: (A) Two-Parameter multiple linear regression for predicting neonicotinoid and transformation product log K_F for nF- (circles) and O-CNTs(diamonds). Gray shapes indicate neonicotinoids used in the training set while white shapes are those in the testing set. Thiacloprid amide is not pictured because no sorption experiments with CNTs were conducted with thiacloprid amide, only GAC sorption was measured for thiacloprid amide (as a fully external validation). (B) Table of the optimal QSAR descriptors (and corresponding coefficients, significance, and multicollinearity [VIF]) for modeling neonicotinoid sorption to nF-/O-CNT (log K_F). This model excludes acetamiprid, thiamethoxam, and thiacloprid amide, which were used in external validation. (C) Experimental GAC log K_F for each neonicotinoid and transformation product (gray) compared to the predicted GAC log K_F. Within the testing set there are two groups: group (a) consisting of acetamiprid and thiamethoxam were externally validated in the CNT multiple linear regression and GAC extrapolation, and group (b) consisting of thiacloprid amide where fully external validation was only conducted following GAC extrapolation.

(A)

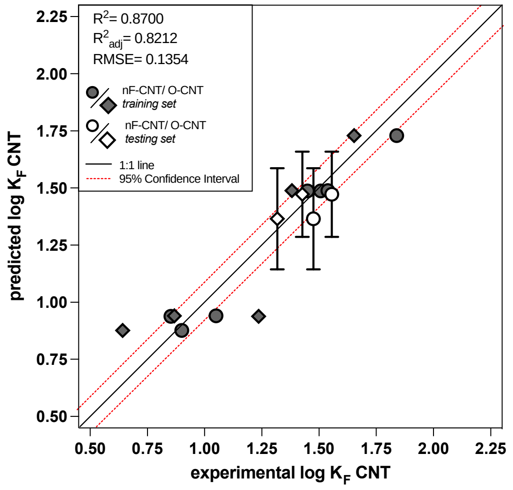

(B)

Descriptor	Coefficient	Standard Error	t-test	p-value	VIF
Intercept	-2.250	0.5049	4.456	0.0021	
Property Min(Surface2) kJ	-0.006939	0.002520	2.754	0.0249	1.825
DBE	0.2172	0.04303	5.048	0.0010	2.178
Property Max(Surface2) kJ	0.002650	0.001275	2.079	0.0713	3.254

R^2= 0.8700, R_{adj}^2= 0.8212, RMSE=0.1354, n=14

(C)

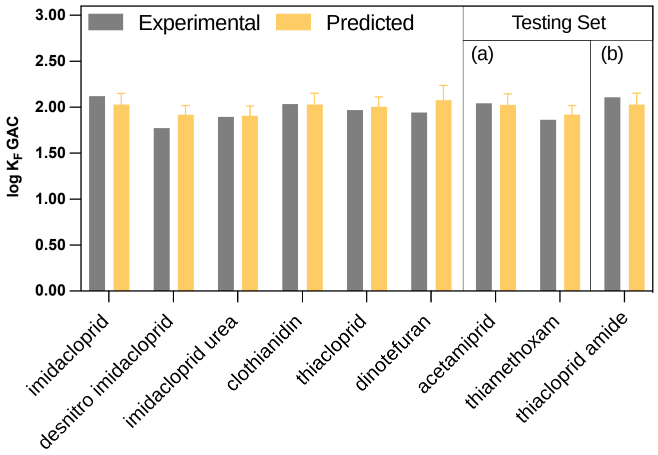

Figure S.11: (A) Three-parameter multiple linear regression for predicting neonicotinoid and transformation product log K_F for nF- (circles) and O-CNTs(diamonds). Gray shapes indicate neonicotinoids used in the training set while white shapes are those in the testing set. Thiacloprid amide is not pictured because no sorption experiments with CNTs were conducted with thiacloprid amide, only GAC sorption was measured for thiacloprid amide (as a fully external validation). (B) Table of the optimal QSAR descriptors (and corresponding coefficients, significance, and multicollinearity [VIF]) for modeling neonicotinoid sorption to nF-/O-CNT (log K_F). This model excludes acetamiprid, thiamethoxam, and thiacloprid amide, which were used in external validation. (C) Experimental GAC log K_F for each neonicotinoid and transformation product (gray) compared to the predicted GAC log K_F. Within the testing set there are two groups: group (a) consisting of acetamiprid and thiamethoxam were externally validated in the CNT multiple linear regression and GAC extrapolation, and group (b) consisting of thiacloprid amide where fully external validation was only conducted following GAC extrapolation.

Table S.20: Predicted water-GAC partition coefficients for neonicotinoid transformation products using two- and three-parameter models. Predictions are presented with 95% confidence intervals

Parent	Transformation Product	Product Structure	Predicted log $K_{F,GAC\text{-}2parameter}$ (min-max)[a]	Predicted log $K_{F,GAC\text{-}3parameter}$ (min-max)[a]
**Imidacloprid**<br>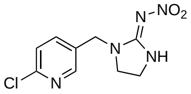<br>Literature log K_{ow}: 0.57<br>Experimental log $K_{F,CNT}$: 1.48<br>Experimental log $K_{F,GAC}$: 2.12	**Imidacloprid olefin**<br>(biodegradation)[b,c]	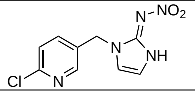	2.12<br>(1.92–2.31)	2.13<br>(1.92–2.20)
	**5-hydroxy imidacloprid**<br>(biodegradation and ozonation)[b,c,d]	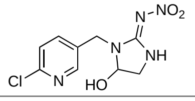	2.00<br>(1.90–2.10)	2.06<br>(1.92-2.20)
	**Imidacloprid 290**<br>(chlorination)[e]	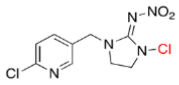	2.04<br>(1.91–2.16)	2.02<br>(1.91–2.14)
**Desnitro-imidacloprid**<br>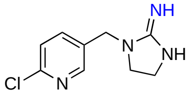<br>Literature log K_{ow}: 0.21<br>Experimental log $K_{F,CNT}$: 1.04<br>Experimental log $K_{F,GAC}$: 1.77	**Desnitro-imidacloprid 245**<br>(chlorination)[e]	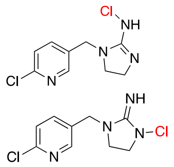	1.86<br>(1.73–2.00)	1.84<br>(1.69–2.00)
	**Desnitro-imidacloprid 279**<br>(chlorination)[e]	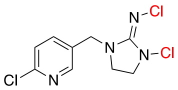	1.86<br>(1.73–2.00)	1.83<br>(1.67–1.99)
**Imidacloprid urea**<br>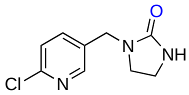<br>Literature log K_{ow}: 0.70<br>Experimental log $K_{F,CNT}$: 0.77<br>Experimental log $K_{F,GAC}$: 1.89	**Imidacloprid urea 246**<br>(chlorination)[e]	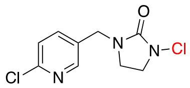	1.96<br>(1.87–2.06)	1.90<br>(1.78–2.01)
**Clothianidin**<br>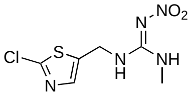<br>Literature log K_{ow}: 0.70<br>Experimental log $K_{F,CNT}$: 1.46<br>Experimental log $K_{F,GAC}$: 2.03	**Clothianidin methyl urea**<br>(biodegradation and hydrolysis)[f,g,h]	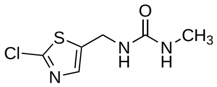	1.91<br>(1.81–2.01)	1.88<br>(1.77–2.00)
	**Clothianidin 239**<br>(chlorination)[e]	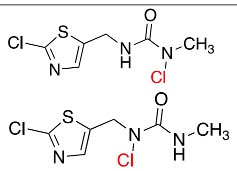	1.85<br>(1.70–1.99)	1.79<br>(1.59–1.99)
	**Clothianidin-Thiamethoxam 270**<br>(chlorination)[e]	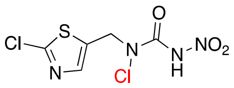	2.00<br>(1.90–2.11)	1.91<br>(1.80–2.01)
**Thiamethoxam**<br>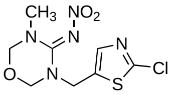<br>Literature log K_{ow}: -0.13<br>Experimental log $K_{F,CNT}$: 1.40<br>Experimental log $K_{F,GAC}$: 1.86	**Thiamethoxam-H 248**<br>(chlorination)[e,h,i,j]	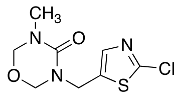	1.89<br>(1.77–2.00)	1.84<br>(1.69–1.99)
	**Thiamethoxam-H 237**<br>(hydrolysis)[e]	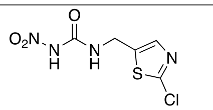	2.02<br>(1.90–2.13)	2.00<br>(1.90–2.10)

(min-max) outlining the range in predicted Freundlich coefficients.

Footnotes: [a]) 95% confidence interval, [b]) Dai et al.[11], [c])Lu et al.[25], [d]) Bourgin et al.[26], [e]) Klarich-Wong et al.[27], [f]) Mulligan et al.[28], [g]) Mori et al.[14], [h]) Todey et al.[29], [i]) Liqing et al.[18], [j]) Pandey et al.[30]

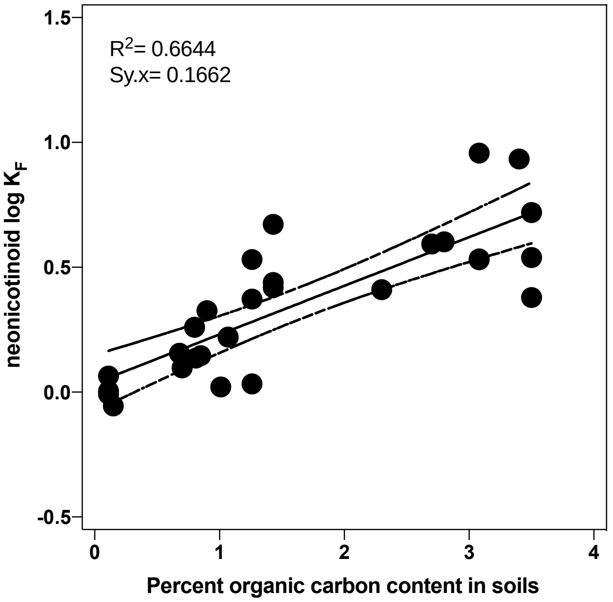

Figure S.12: Correlation between soil organic carbon content and Freundlich constant (log K_F) for neonicotinoids (imidacloprid, clothianidin, thiamethoxam, thiacloprid, acetamiprid) based on literature values.[31]

References

(1) Webb, D. T.; Nagorzanski, M. R.; Powers, M. M.; Cwiertny, D. M.; Hladik, M. L.; LeFevre, G. H. Differences in Neonicotinoid and Metabolite Sorption to Activated Carbon Are Driven by Alterations to the Insecticidal Pharmacophore. *Environ. Sci. Technol.* **2020**, *54* (22), 14694–14705. https://doi.org/10.1021/acs.est.0c04187.

(2) Peter, K. T.; Vargo, J. D.; Rupasinghe, T. P.; De Jesus, A.; Tivanski, A. V; Sander, E. A.; Myung, N. V; Cwiertny, D. M. Synthesis, Optimization, and Performance Demonstration of Electrospun Carbon Nanofiber–Carbon Nanotube Composite Sorbents for Point-of-Use Water Treatment. *ACS Appl. Mater. Interfaces* **2016**, *8* (18), 11431–11440. https://doi.org/10.1021/acsami.6b01253.

(3) Tomizawa, M.; Zhang, N.; Durkin, K. A.; Olmstead, M. M.; Casida, J. E. The Neonicotinoid Electronegative Pharmacophore Plays the Crucial Role in the High Affinity and Selectivity for the Drosophila Nicotinic Receptor: An Anomaly for the Nicotinoid Cation--Pi Interaction Model. *Biochemistry* **2003**, *42* (25), 7819–7827. https://doi.org/10.1021/bi0300130.

(4) Tülp, H. C.; Goss, K.-U.; Schwarzenbach, R. P.; Fenner, K. Experimental Determination of LSER Parameters for a Set of 76 Diverse Pesticides and Pharmaceuticals. *Environ. Sci. Technol.* **2008**, *42* (6), 2034–2040. https://doi.org/10.1021/es702473f.

(5) Abraham, M. H.; McGowan, J. C. The Use of Characteristic Volumes to Measure Cavity Terms in Reverse Phase Liquid Chromatography. *Chromatographia* **1987**, *23* (4), 29–47.

(6) McGowan, J. C.; Mellors, A. *Molecular Volumes in Chemistry and Biology: Applications Including Partitioning and Toxicity*; Ellis Horwood Chichester, 1986.

(7) Zhao, Y. H.; Abraham, M. H.; Zissimos, A. M. Determination of McGowan Volumes for Ions and Correlation with van Der Waals Volumes. *J. Chem. Inf. Comput. Sci.* **2003**, *43* (6), 1848–1854. https://doi.org/10.1021/ci0341114.

(8) Klarich, K. L.; Pflug, N. C.; DeWald, E. M.; Hladik, M. L.; Kolpin, D. W.; Cwiertny, D. M.; LeFevre, G. H. Occurrence of Neonicotinoid Insecticides in Finished Drinking Water and Fate during Drinking Water Treatment. *Environ. Sci. Technol. Lett.* **2017**, *4* (5), 168–173. https://doi.org/10.1021/acs.estlett.7b00081.

(9) Klarich Wong, K. L.; T. Webb, D.; R. Nagorzanski, M.; W. Kolpin, D.; L. Hladik, M.; M. Cwiertny, D.; H. LeFevre, G. Chlorinated Byproducts of Neonicotinoids and Their Metabolites: An Unrecognized Human

Exposure Potential? *Environ. Sci. & Technol. Lett.* **2019**, *6* (2), 98–105. https://doi.org/10.1021/acs.estlett.8b00706.
(10) APHA; AWWA; WEF. *Standard Methods for the Examination of Water and Wastewater*; 1999.
(11) Dai, Y.; Yuan, S.; Ge, F.; Chen, T.; Xu, S.; Ni, J. Microbial Hydroxylation of Imidacloprid for the Synthesis of Highly Insecticidal Olefin Imidacloprid. *Appl. Microbiol. Biotechnol.* **2006**, *71* (6), 927–934. https://doi.org/10.1007/s00253-005-0223-3.
(12) Mulligan, R. A.; Tomco, P. L.; Howard, M. W.; Schempp, T. T.; Stewart, D. J.; Stacey, P. M.; Ball, D. B.; Tjeerdema, R. S. Aerobic versus Anaerobic Microbial Degradation of Clothianidin under Simulated California Rice Field Conditions. *J. Agric. Food Chem.* **2016**, *64* (38), 7059–7067. https://doi.org/10.1021/acs.jafc.6b02055.
(13) Lu, T. Q.; Mao, S. Y.; Sun, S. L.; Yang, W. L.; Ge, F.; Dai, Y. J. Regulation of Hydroxylation and Nitroreduction Pathways during Metabolism of the Neonicotinoid Insecticide Imidacloprid by Pseudomonas Putida. *J. Agric. Food Chem.* **2016**, *64* (24), 4866–4875. https://doi.org/10.1021/acs.jafc.6b01376.
(14) Mori, T.; Wang, J.; Tanaka, Y.; Nagai, K.; Kawagishi, H.; Hirai, H. Bioremediation of the Neonicotinoid Insecticide Clothianidin by the White-Rot Fungus Phanerochaete Sordida. *J. Hazard. Mater.* **2017**, *321*, 586–590. https://doi.org/http://dx.doi.org/10.1016/j.jhazmat.2016.09.049.
(15) Pandey, G.; Dorrian, S. J.; Russell, R. J.; Oakeshott, J. G. Biotransformation of the Neonicotinoid Insecticides Imidacloprid and Thiamethoxam by Pseudomonas Sp. 1G. *Biochem. Biophys. Res. Commun.* **2009**, *380* (3), 710–714. https://doi.org/10.1016/j.bbrc.2009.01.156.
(16) Todey, S. A.; Fallon, A. M.; Arnold, W. A. Neonicotinoid Insecticide Hydrolysis and Photolysis: Rates and Residual Toxicity. *Environ. Toxicol. Chem.* **2018**, *37* (11), 2797–2809. https://doi.org/10.1002/etc.4256.
(17) Klarich Wong, K. L.; Webb, D. T.; Nagorzanski, M. R.; Kolpin, D. W.; Hladik, M. L.; Cwiertny, D. M.; Lefevre, G. H. Chlorinated Byproducts of Neonicotinoids and Their Metabolites: An Unrecognized Human Exposure Potential? *Environ. Sci. Technol. Lett.* **2019**, *6* (2). https://doi.org/10.1021/acs.estlett.8b00706.
(18) Liqing, Z.; Guoguang, L.; Dezhi, S.; Kun, Y. Hydrolysis of Thiamethoxam. *Bull. Environ. Contam. Toxicol.* **2006**, *76* (6), 942–949. https://doi.org/10.1007/s00128-006-1009-8.
(19) Goss, K.-U.; Schwarzenbach, R. P. Linear Free Energy Relationships Used To Evaluate Equilibrium Partitioning of Organic Compounds. *Environ. Sci. Technol.* **2001**, *35* (1), 1–9. https://doi.org/10.1021/es000996d.
(20) Schwarzenbach, R.; Gschwend, P.; Imdoden, D. *Environmental Organic Chemistry*; John wiley & Sons: New Jersey, 2003; Vol. Second. https://doi.org/10.1002/0471649643.
(21) McEachran, A. D.; Sobus, J. R.; Williams, A. J. Identifying Known Unknowns Using the US EPA's CompTox Chemistry Dashboard. *Anal. Bioanal. Chem.* **2017**, *409* (7), 1729–1735. https://doi.org/10.1007/s00216-016-0139-z.
(22) Williams, A. J.; Grulke, C. M.; Edwards, J.; McEachran, A. D.; Mansouri, K.; Baker, N. C.; Patlewicz, G.; Shah, I.; Wambaugh, J. F.; Judson, R. S.; Richard, A. M. The CompTox Chemistry Dashboard: A Community Data Resource for Environmental Chemistry. *J. Cheminform.* **2017**, *9* (1). https://doi.org/10.1186/s13321-017-0247-6.
(23) Sigmund, G.; Gharasoo, M.; Hüffer, T.; Hofmann, T. Deep Learning Neural Network Approach for Predicting the Sorption of Ionizable and Polar Organic Pollutants to a Wide Range of Carbonaceous Materials. *Environ. Sci. Technol.* **2020**, *54* (7), 4583–4591. https://doi.org/10.1021/acs.est.9b06287.
(24) Selmi, T.; Seffen, M.; Sammouda, H.; Mathieu, S.; Jagiello, J.; Celzard, A.; Fierro, V. Physical Meaning of the Parameters Used in Fractal Kinetic and Generalised Adsorption Models of Brouers–Sotolongo. *Adsorption* **2018**, *24* (1), 11–27. https://doi.org/10.1007/s10450-017-9927-9.
(25) Lu, T.-Q.; Mao, S.-Y.; Sun, S.-L.; Yang, W.-L.; Ge, F.; Dai, Y.-J. Regulation of Hydroxylation and Nitroreduction Pathways during Metabolism of the Neonicotinoid Insecticide Imidacloprid by Pseudomonas Putida. *J. Agric. Food Chem.* **2016**, *64* (24), 4866–4875. https://doi.org/10.1021/acs.jafc.6b01376.
(26) Bourgin, M.; Violleau, F.; Debrauwer, L.; Albet, J. Ozonation of Imidacloprid in Aqueous Solutions: Reaction Monitoring and Identification of Degradation Products. *J. Hazard. Mater.* **2011**, *190* (1–3), 60–68. https://doi.org/10.1016/j.jhazmat.2011.02.065.
(27) Klarich-Wong, K. L.; Webb, D. T.; Nagorzanski, M. R.; Kolpin, D. W.; Hladik, M. L.; Cwiertny, D. M.; LeFevre, G. H. Chlorinated Byproducts of Neonicotinoids and Their Metabolites: An Unrecognized Human Exposure Potential? *Environ. Sci. Technol. Lett.* **2019**, *6* (2), 98–105. https://doi.org/10.1021/acs.estlett.8b00706.
(28) Mulligan, R. A.; Tomco, P. L.; Howard, M. W.; Schempp, T. T.; Stewart, D. J.; Stacey, P. M.; Ball, D. B.; Tjeerdema, R. S. Aerobic versus Anaerobic Microbial Degradation of Clothianidin under Simulated California

Rice Field Conditions. *J. Agric. Food Chem.* **2016**, *64* (38), 7059–7067. https://doi.org/10.1021/acs.jafc.6b02055.

(29) Todey, S. A.; Fallon, A. M.; Arnold, W. A. Neonicotinoid Insecticide Hydrolysis and Photolysis: Rates and Residual Toxicity. *Environ. Toxicol. Chem.* **2018**, *37* (11), 2797–2809. https://doi.org/10.1002/etc.4256.

(30) Pandey, G.; Dorrian, S. J.; Russell, R. J.; Oakeshott, J. G. Biotransformation of the Neonicotinoid Insecticides Imidacloprid and Thiamethoxam by Pseudomonas Sp. 1G. *Biochem. Biophys. Res. Commun.* **2009**, *380* (3), 710–714. https://doi.org/http://dx.doi.org/10.1016/j.bbrc.2009.01.156.

(31) Pietrzak, D.; Kania, J.; Kmiecik, E.; Malina, G.; Wątor, K. Fate of Selected Neonicotinoid Insecticides in Soil–Water Systems: Current State of the Art and Knowledge Gaps. *Chemosphere* **2020**, *255*, 126981. https://doi.org/10.1016/j.chemosphere.2020.126981.
